# Understanding traumatic brain injury in elderly populations: a developing country perspective

**DOI:** 10.1016/j.clinsp.2026.101054

**Published:** 2026-07-28

**Authors:** Gabriel de Moraes Ribeiro, Matheus Ballestero, Leandro Candido de Souza, Lucas Rodrigues Olmedo, Rodrigo Inácio Pongeluppi, Benedicto Oscar Colli, Ricardo Santos de Oliveira

**Affiliations:** a Division of Neurosurgery, Ribeirao Preto School of Medicine, University of Sao Paulo, Ribeirão Preto, Sao Paulo, Brazil; b Department of Medicine, Universidade Federal de São Carlos (UFSCar), São Carlos, SP, Brazil

**Keywords:** Epidemiology, Neurosurgery, Traumatic brain injury, Geriatric

## Abstract

**Background:**

With the steady increase in life expectancy over recent decades, elderly healthcare has become a global priority. Traumatic Brain Injuries (TBIs) in older adults pose a significant public health challenge due to the increased susceptibility of this age group to such injuries.

**Objective:**

This study aims to elucidate the epidemiological patterns of Traumatic Brain Injury (TBI) in elderly individuals in a developing country, focusing on incidence, outcomes, and associated costs.

**Methods:**

This retrospective cross-sectional study included 1268 elderly TBI patients at a Brazilian tertiary trauma center from 2010 to 2022. Variables included age, gender, injury mechanism, hospitalization details, and outcomes. Logistic regression analyzed predictors of outcome.

**Results:**

A total of 1268 elderly patients with TBI were initially screened; 93 were excluded, yielding a final analytic sample of 1175 patients. 1049 (89.3%) TBIs were classified as mild, with falls being the predominant cause of TBI, appearing in 904 cases (76.9%). The average annual hospitalization rate for elderly TBI patients was 90 cases. Significant predictors of unfavorable outcomes included age, low education levels, and intracranial injuries. The study also emphasizes costs associated with TBI care as a substantial economic burden.

**Conclusions:**

The study highlights the rising incidence and economic burden of TBI in elderly populations in developing countries, emphasizing the need for targeted public health policies and resource allocation to address this growing issue.

## Highlights

• Falls were the main cause of TBI in the elderly, accounting for 76.9% of cases.
• Age > 80 years and low education predicted worse functional outcomes.
• Intracranial injuries increased mortality compared to extracranial injuries.
• Average cost per patient was US$1152, rising during the pandemic period.
• Findings highlight urgent need for policies to prevent TBI in the elderly.

## Introduction

Elderly healthcare has become a priority, given the progressive increase in life expectancy observed in recent decades. The number of individuals aged over 65-years was 129 million in 1950, which rose to 771 million in 2022 and is projected to reach 2.5 billion in 2100.^1^

From the perspective of the country's aging population, elderly healthcare must increasingly become a higher priority. In recent years, trauma prevalence among elderly individuals has risen significantly.^2^ Traumatic Brain Injuries (TBIs) in this demographic represent a major public health concern, largely due to common vulnerabilities in this stage of life.^3^ However, few studies address this issue, hindering the development of effective public policies to mitigate this growing social problem.

Recent and updated data provided by DATASUS (Brazilian Public Health Care Database) for 2010 through 2023 show an average of 19,624 hospitalizations per year in patients aged 65 and older, with a particular emphasis on victims over 80-years (34% of all hospitalizations). Furthermore, the data demonstrate a notable upward trend over time, with 13,112 hospitalizations recorded in 2010 and 25,338 in 2023. The average mortality rate ranged from 13.73% to 19.54% across the different age groups, with a total average of 16.33%. The total expenditure on the care of these victims in 2023 was 12,582,000.00 dollars.^4^

Given the rising life expectancy and demographic shifts in developing countries, which exacerbate the social and economic challenges associated with TBIs in the elderly, alongside the scarcity of regional data, this study aims to clarify the epidemiological patterns of TBI in individuals aged 65 and older, providing critical insights for future health policy development.

## Materials and methods

This is a cross-sectional study that involves retrospective data collection from individuals aged 65-years and older who have suffered from TBI and were treated at the Emergency Department of a tertiary trauma center in Brazil. It received approval from the local Research Ethics Committee, the requirement for individual informed consent was waived by the committee due to the retrospective nature of the data analysis.

The study included individuals diagnosed with mild, moderate, or severe TBI treated at the Emergency Department of University Hospital of the Medical School of Ribeirão Preto (HCFMRP-USP) between 2010 and 2022 and aged 65-years or older. Patients with uncertain or unavailable clinical outcomes at discharge and those with incomplete data were excluded from the study.

The independent variables studied were age; gender; education level; ethnicity (white, black, Asian, or mixed); occupation; mechanism of injury (motor vehicle, motorcyclist, pedestrian, cyclist, stabbing, firearm, fall, burn, machinery accident, assault, or other); days of hospitalization in the Intensive Care Unit (ICU); performance of surgery; number of surgeries performed; Glasgow Coma Scale (GCS) score; and etiology of injury, listed according to ICD-10 classification codes (S00‒S09 as primary injury, and all the others as secondary injury). The etiologies of primary injury were further regrouped into intracranial injuries (S060‒S069) and extracranial injuries (S00‒S09, with the exception of S06).

The dependent variables were the Glasgow Outcome Scale (GOS) score and mortality.

To assess the impact of each independent variable on the outcome, the authors first conducted a univariable logistic regression analysis to identify significant associations. Only variables with statistical significance (p < 0.05) in the univariable analyses were then included in a binary logistic regression model to evaluate their combined effect, accounting for interactions and confounders, as indicated by odds ratios. Age, a common confounder, was addressed using the Area Under the receiver operating Characteristic Curve (AUC) to compare model performance with and without this variable, ensuring the quantification of its predictive impact and reducing bias.

Missing data were addressed using complete case analysis. Only patients with available data for all variables included in univariable and multivariable primary analysis were retained. To assess the impact of excluding 93 patients (7.3% of the initial sample) due to missing data, a three-step sensitivity analysis was conducted:

1- Characterization of excluded patients: Available variables (age, sex, trauma mechanism, and GCS) were compared between included and excluded patients using chi-square tests for categorical variables and Student’s *t*-test or Mann-Whitney tests for continuous variables.
2- Extreme-case scenarios: It was alternatively assumed that all excluded patients had either a favorable functional outcome (GOS 5) or an unfavorable outcome (GOS 1–4). In each scenario, outcome proportions were recalculated, and original logistic regression models (univariate and multivariate) were re-estimated.
3- Multiple imputation: For excluded patients with partial predictor data, Multiple Imputation by Chained Equations (MICE) was performed with 20 imputed datasets. Regression models were re-run, and final estimates were combined using Rubin’s rules.

Differences between the original models and those obtained in the sensitivity analyses were evaluated by comparing the magnitude and direction of Odds Ratios (ORs), 95% confidence intervals, and the Area Under the ROC Curve (AUC).

The cost of medical services was calculated based on three main components: the number of days spent in the hospital ward, the number of days spent in the ICU, and the cost of surgical procedures. Unit cost data for each component were obtained directly from the hospital’s administrative and financial accounting records. These values represent actual hospital expenditures rather than standardized reimbursement tables or government-defined estimates. The administrative department calculated costs using a mean-based allocation approach, in which the total institutional expenditure for each cost category during the study period was divided by the corresponding unit of analysis (e.g., total ward-days, ICU-days, or surgical hours). This method reflects the average real financial burden incurred by the hospital in providing TBI-related care. Detailed annual unit costs are provided in the Supplementary Table. All cost values from 2010 to 2022 were adjusted for inflation using the official Brazilian Consumer Price Index (*Índice Nacional de Preçosao Consumidor Amplo* – IPCA) published by the *Instituto Brasileiro de Geografia e Estatística* (IBGE). This adjustment allowed comparability across years by expressing values in constant 2022 Reais (BRL). After inflation adjustment, total costs were converted into U.S. Dollars (USD) using the May 2022 average annual exchange rate of 5.07 BRL per USD, according to data from the Brazilian Central Bank.

In order to assess the impact of the SARS-CoV-2 virus pandemic on the epidemiological profile of TBIs, data on the incidence of COVID-19 in the trauma center health macro-region were obtained from the CORONAVÍRUS BRASIL platform. To assess the effect of the COVID-19 pandemic on TBI incidence and outcomes, the authors conducted an Interrupted Time Series (ITS) analysis covering the period from January 2019 to December 2021. The intervention point was set as March 2020, when the first lockdown was declared in the State of São Paulo. Monthly TBI incidence rates were modeled using Ordinary Least Squares with Heteroskedasticity- and Autocorrelation-Consistent (OLS-HAC) standard errors, as well as Generalized Linear Models (GLM) with a Poisson distribution for count data. Models included adjustments for monthly COVID-19 incidence, mobility restriction indices, and the total volume of hospital admissions to account for potential confounding due to healthcare system strain and changes in care-seeking behavior. The authors estimated both the immediate change in incidence following lockdown (level change, β_post) and the change in monthly trend thereafter (slope change, β_time_post). Outcome models assessed the association between COVID-19 incidence and unfavorable functional outcomes (GOS 1–4) and mortality before and after adjustment for confounders.

This study was conducted and reported in accordance with the STROBE (Strengthening the Reporting of Observational Studies in Epidemiology) guidelines for cross-sectional studies.

## Results

### Study population

From January 2010 to December 2022, 1268 individuals were initially identified for inclusion in the study. Among these patients, 21 (1.7%) were excluded because of a lack of TBI history, and 72 (5.6%) were excluded because of uncertain discharge outcomes or unobtainable data, with a total of 1175 final participants retained.

### Severity and outcomes of TBI

In the period under review, the number of TBI cases ranged from 33 in 2013 to 140 in 2022, with an average annual rate of 90 cases (σ = 37.66). Among the cases, 1049 (89.3%) were classified as mild according to the Glasgow Coma Scale (GCS) 13‒15, whereas only 125 (9.1%) were classified as severe GCS 3‒8.

With respect to discharge status, the percentage of patients with unfavorable outcomes, defined as Glasgow Outcome Scale (GOS) scores of 1‒4, was 675 (57.4%), some of whom died 135 times (11.5%).

### Injury

A review of the primary injuries among TBI patients revealed that the most prevalent injuries were classified as extracranial. These included head injuries (S01), fractures of the skull and facial bones (S02), dislocations, sprains or strains of the joints and ligaments of the head (S03), trauma to the eye and orbit (S05), traumatic amputation of part of the head (S08) and other unspecified head injuries (S09), accounting for a total of 746 cases (63.48%). The remaining were intracranial injuries, with subdural hemorrhage (S065) being the most prevalent, present in 106 cases (9.00%), followed by cerebral concussion (S060), with 72 cases (6.10%). Additionally, focal brain trauma (S063) was observed in 54 patients (4.60%), and traumatic cerebral edema (S061) was found in 53 patients (4.50%) (Table 1).

**Table 1:** Distribution of primary and secondary injuries and univariable logistic regression for functional outcome (GOS) significance.

Primary Injury	n	Sig for Functional Outcome (GOS)	Sig for Mortality
Extracranial Injuries	746 (63.48%)	p < 0.001	p < 0.001
Subdural hemorrhage	106 (9.00%)	p < 0.001	p < 0.001
Cerebral concussion	72 (6.10%)	p < 0.001	p < 0.001
Focal brain trauma	54 (4.60%)	p < 0.001	p = 0.002
Traumatic cerebral edema	53 (4.50%)	p = 0.021	p = 0.018
Traumatic subarachnoid hemorrhage	44 (3.80%)	p < 0.001	p < 0.001
Others Intracranial Injuries	32 (2.72%)	p = 0.002	p = 0.009
Epidural hematoma	25 (2.10%)	p < 0.001	p < 0.001
Diffuse brain trauma	22 (1.90%)	p = 0.003	p = 0.071
Intracranial trauma with prolonged coma	20 (1.70%)	p = 0.007	p = 0.001
**Secondary Injury**	**n**	**Sig for Functional Outcome (GOS)**	**Sig for Mortality**
Thorax	66 (5.6%)	p = 0.254	p = 0.305
Abdomen, Lumbar, and Pelvis	54 (4.6%)	p = 0.058	p = 0.066
Upper Limbs	79 (6.7%)	p = 0.641	p = 0.978
Lower Limbs	64 (5.4%)	p = 0.327	p = 0.585
Other Injuries	43 (3.7%)	p = 0.358	p = 0.344

With regard to secondary injuries, the upper limbs were the most affected anatomical segment, with 79 cases (6.7%), followed by the thorax with 66 cases (5.6%) and the lower limbs with 64 cases (5.4%). Abdominal, lumbar, and pelvic injuries were documented in 54 cases (4.6%). Multiple injuries, burns and other less common injuries were relatively uncommon, collectively accounting for 43 cases (3.7%) (Table 1).

### Demography and epidemiology

Most of the TBI victims treated were over 80-years-old, n = 376 (32%), and white, n = 1023 (87.1%). There was no significant difference between males, n = 606 (51.6%) and females, n = 569 (48.4%). Most victims had a medium level of education, particularly primary education, registered in 619 patients (52.7%), and were economically inactive, n = 952 (81.1%) (Table 2).

**Table 2:** Epidemiological profile.

Categories	Subcategories	Frequency and Percentage	Sig for Functional Outcome (GOS 1‒4)	Sig for Mortality
Age	65‒69	327 (27.8%)	p = 0.022	p = 0.002
	70‒74	255 (21.7%)	p = 0.024	p = 0.001
	75‒79	217 (18.5%)	p = 0.326	p = 0.008
	80+	376 (32.0%)	p = 0.021	p = 0.001
Total		1175 (100%)		
Education	Low	453 (38.6%)	p = 0.004	p = 0.002
	Medium	619 (52.7%)	p = 0.402	p = 0.001
	High	103 (8.8%)	p < 0.001	p < 0.001
Total		1175 (100%)		
Ethnicity	White	1023 (87.1%)	p = 0.289	p = 0.180
	Black	54 (4.6%)	p = 0.439	p = 0.428
	Asian	10 (0.9%)	p = 0.432	p = 0.999
	Mixed	88 (7.4%)	p = 0.154	p = 0.058
Total		1175 (100%)		
Occupation	Not informed	15 (1.3%)	p = 0.678	p = 0.875
	Active	208 (17.7%)	p = 0.431	p = 0.571
	Retired	760 (64.7%)	p = 0.625	p = 0.185
	Homemaker	192 (16.3%)	p = 0.525	p = 0.379
Total		1175 (100%)		
Sex	Male	606 (51.6%)	p = 0.518	p = 0.244
	Female	569 (48.4%)	p = 0.518	p = 0.244
Total		1175 (100%)		
Mechanism of Injury	Traffic	149 (12.7%)	p = 0.045	p = 0.006
	Aggression	48 (4.1%)	p = 0.488	p = 0.212
	Others	74 (6.3%)	p = 0.064	p = 0.863
	Falls	904 (76.9%)	p = 0.434	p = 0.014
Total		1175 (100%)		

Note: Description of categorical variables by absolute and relative frequencies. Relationship of each subcategory to functional outcome according to GOS, expressed as prevalence ratio. Percentages are calculated based on the total sample of n = 1175. The p-values represent the univariable logistic regression for Functional Outcome (GOS 1‒4) significance of each subcategory.

Falls were the leading cause of TBI, accounting for 904 cases (76.9%). Road traffic accidents involving pedestrians or drivers/passengers ranked second with 149 cases (12.7%), followed by TBI resulting from a physical assault, with 48 cases (4.1%). The remaining 74 cases (6.3%) were associated with other causes, including accidents at work or domestic and wild animals, or were not reported (Table 2).

### Economic analysis

The direct costs of treating TBI patients were estimated on the basis of the data in Table 2. A total of 218 surgeries were performed, with an average duration of 3.2 hours (σ = 0.266). Patients were hospitalized for a total of 3990 days in ordinary wards and 516-days in the ICU. Consequently, the total estimated cost was $1353,723.62, with an annual average of $104,132.59. The average cost per patient was $1152.11, ranging from $476.19 in 2010 to $1932.49 in 2021 (Table 3).

**Table 3:** Costs of care.

	Average Ward-Day Cost (USD)	Average ICU-Day Cost (USD)	Surgical Hour Cost (USD)	Average Quotation (USD/BRL)
**2010**	399.01	1325.47	661.34	1.7521
**2011**	485.03	1575.61	795.69	1.6707
**2012**	546.31	1723.76	809.98	1.9603
**2013**	582.63	1938.35	514.01	2.2089
**2014**	669.37	2039.30	673.78	2.3536
**2015**	818.88	2132.46	824.00	3.3338
**2016**	1016.16	1772.43	804.78	3.4869
**2017**	909.04	1946.08	814.6	3.1916
**2018**	1081.84	2124.79	967.99	3.6551
**2019**	1095.66	2165.50	920.04	3.9563
**2020**	962.94	2335.33	625.4	5.1969
**2021**	953.94	2308.31	561.43	5.3656
**2022**	1161.53	2515.69	614.94	5.1627

Note: This table presents the annual cost of the daily cost of hospitalization in a surgery ward, the daily cost of hospitalization in an ICU and a surgical hour, in the Emergency Unit of the *Hospital das Clínicas de Ribeirão Preto*, and the average annual dollar/real exchange rate from 2010‒2022.

### Inferential statistics

Univariable logistic regression for functional outcome (GOS) significance revealed that the type of primary injury, age and trauma mechanism were predictors of an unfavorable outcome (GOS score 1‒4 and mortality) (Table 1, Table 2). Other Sociodemographic variables such as ethnicity and occupation were excluded from the final models due to lack of statistical significance in the univariable analysis.

**Fig. 1:**
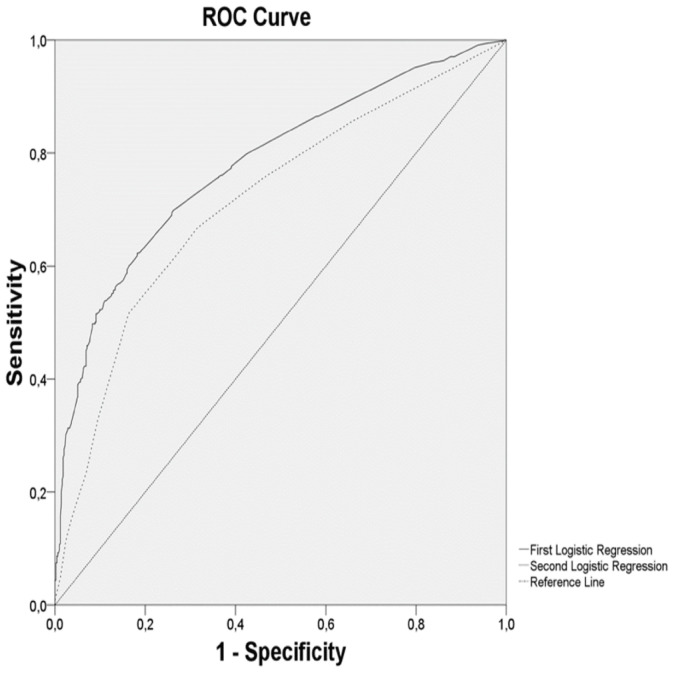
The comparative Receiver Operating characteristic (ROC) curve of two binary logistic regression models with Glasgow Outcome Scale (GOS) 1‒4 as the dependent variables presented. The first model incorporates all the categorical variables (age, education, mechanism of injury, and etiology of injury), while the second model includes these variables, with the exception of age. AUC of First Logistic Regression = 0.782 and AUC of Second Logistic Regression = 0.728.

**Fig. 2:**
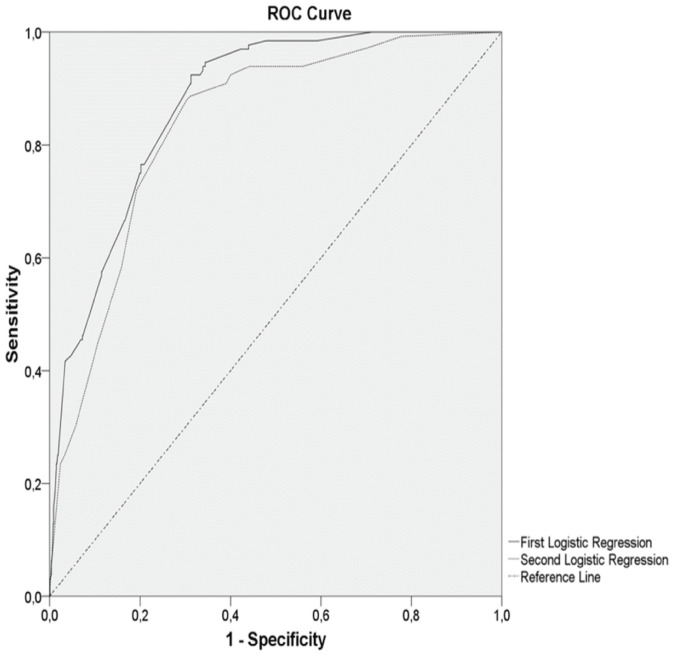
The comparative Receiver Operating Characteristic (ROC) curve of two binary logistic regression models with mortality as the dependent variable is present. The first model incorporates all the categorical variables (age, education, mechanism of injury, and etiology of injury), while the second model includes these variables, with the exception of age. AUC of First Logistic Regression = 0.886 and AUC of Second Logistic Regression = 0.842.

Binary logistic regression models identified predictors of unfavorable outcomes (GOS 1‒4 and mortality). The model for GOS 1‒4 was significant [X^2^(1) = 182.322; p < 0.001, R^2^ Nagelkerke = 0.245], as was the mortality model [X^2^(1) = 268.473; p < 0.001, R^2^ Nagelkerke = 0.401]. Including all categorical variables yielded AUCs of 0.782 (GOS 1‒4) and 0.886 (mortality). Without age, AUCs decreased to 0.728 and 0.842 (Fig. 1, Fig. 2).

Non-intracranial injuries had a greater proportion of GOS 5 results. Specifically, 414 patients had this outcome, representing 55.49% of the 746 patients with these injuries. In this context, the relative risk of the subcategories of intracranial injuries developing GOS scores of 1‒4 compared with those of extracranial injuries was verified. Injuries classified as subdural hemorrhage (S065) had an OR for GOS 1‒4 of 11.887 (95% CI: 5.888, 23.996), focal brain trauma (S063) had an OR of 13.396 (95% CI: 4.699, 38.184) for GOS 1‒4, whereas traumatic cerebral edema (S061) had an OR of 5.587 (95% CI: 2.740, 11.393), and traumatic subarachnoid hemorrhage (S066) had an OR of 9.783 (95% CI: 3.785, 25.283). The intracranial injury that had the lowest Odds Ratio (OR) was epidural hematoma (S064), with an OR of 2.184 (95% CI: 1.056, 4.517). The intracranial injuries with the highest risk of death were subarachnoid hemorrhage due to trauma and subdural hemorrhage due to trauma, with ORs of 27.728 (95% CI: 14.198, 54.151) and 29.010 (95% CI: 12.085, 69.637), respectively (Table 4).

**Table 4:** multivariable logistic regression analysis of primary injury subtypes as predictors of unfavorable functional outcome (GOS 1–4) and mortality.

Primary injury	GOS 1‒4				Mortality			
	Sig	OR	95% CI para OR		Sig	OR	95% CI para OR	
			Lower	Uper			Lower	Uper
Extracranial injuries (ref)*	‒	‒	‒	‒	‒	‒	‒	‒
Cerebral concussion	<0.001	2.257	1.351	3.770	<0.001	11.409	4.998	26.045
Traumatic cerebral edema	<0.001	5.587	2.740	11.393	<0.001	20.769	9.037	47.735
Diffuse brain trauma	<0.001	3.487	1.335	9.109	<0.001	12.209	3.316	44.943
Focal brain trauma	<0.001	13.396	4.699	38.184	<0.001	17.542	7.240	42.499
Epidural hemorrhage	0.035	2.184	1.056	4.517	0.004	6.046	1.767	20.686
Subdural hemorrhage dueto trauma	<0.001	11.887	5.888	23.996	<0.001	27.728	14.198	54.151
Subarachnoid hemorrhage dueto trauma	<0.001	9.783	3.785	25.283	<0.001	29.010	12.085	69.637
Intracranial trauma with prolonged coma	<0.001	13.156	2.987	57.940	<0.001	18.737	5.666	61.961
Other intracranial injuries	<0.001	6.247	2.326	16.777	<0.001	23.905	8.915	64.104

Note: This table presents the results of a multivariable logistic regression model evaluating the association between primary injury patterns and clinical outcomes (GOS 1–4 and mortality), with extracranial injuries as the reference category. Odds Ratios (ORs) are reported with 95% Confidence Intervals (95% CI) and corresponding p-values.

Regarding sociodemographic factors, older age (80-years or above) was more significantly associated with worse GOS outcomes, representing 35.1% of individuals classified as GOS 1‒4, with an OR of 1.388 (95% CI: 1.054‒1.828) compared to younger age groups. In contrast, younger individuals had a lower risk than those over 80; the OR for individuals aged 65‒69 was 0.678 (95% CI: 0.482‒0.953), and for those aged 70‒74, it was 0.627 (95% CI: 0.437‒0.899). Similarly, individuals with low education (incomplete primary education) were more strongly associated with poorer GOS outcomes, with an OR of 1.918 (95% CI: 1.194‒3.081), while those with higher education showed a lower association with adverse outcomes, with an OR of 0.562 (95% CI: 0.362‒0.873). No significant associations were found between sex, ethnicity, or occupation and functional outcomes. These trends were more pronounced for mortality, with individuals over 80 and those with lower education levels at higher risk of death (Table 5).

**Table 5:** Multivariable logistic regression analysis of sociodemographic variables and mechanism of injury as predictors of unfavorable functional outcome (GOS 1–4) and mortality.

	GOS 1‒4				Mortality			
	Sig	OR	95% CI para OR		Sig	OR	95% CI para OR	
			Lower	Uper			Lower	Uper
**Age (years old)**								
80+ (ref)**	‒	‒	‒	‒	‒	‒	‒	‒
65‒69 (ref: ≥ 80)	0.026	0.678	0.482	0.953	0.002	0.421	0.241	0.737
70‒74 (ref: ≥ 80)	0.011	0.627	0.437	0.899	<0.001	0.323	0.173	0.602
75‒79 (ref: ≥ 80)	0.285	0.815	0.560	1.186	0.009	0.428	0.226	0.810
**Education**								
Low (ref)***	‒	‒	‒	‒	‒	‒	‒	‒
Moderate	0.038	1.626	1.027	2.574	0.471	0.750	0.342	1.641
High	0.010	0.562	0.362	0.873	0.011	0.363	0.166	0.791
**Mechanism of injury**								
Falls****	‒	‒	‒	‒	‒	‒	‒	‒
Traffic	0.030	1.500	1.040	2.161	0.025	2.865	1.143	7.181
Aggression	0.836	0.929	0.463	1.865	0.392	1.316	0.702	2.468
Others	0.054	0.589	0.344	1.010	0.246	1.698	0.695	4.149
**Age (years old)**								
65‒79	‒	‒	‒	‒				
80+ (ref: 65‒79)**	0.019	1.388	1.054	1.828	<0.001	2.489	1.613	3.842
**Education**								
Moderate + High	‒	‒	‒	‒	‒	‒	‒	‒
Low***	0.007	1.918	1.194	3.081	0.002	1.941	1.278	2.950
**Mechanism of injury**								
Non falls	‒	‒	‒	‒	‒	‒	‒	‒
Falls****	0.881	0.977	0.722	1.322	0.028	0.592	0.371	0.945

Note: This table presents the results of two complementary multivariable logistic regression models assessing the association between social demographic factors and mechanism injury with unfavorable functional outcome (GOS 1–4) and mortality. Results are expressed as Odds Ratios (ORs) with 95% Confidence Intervals (95% CI) and corresponding p-values.

In the first model, the reference categories were age ≥80-years, low educational level, and falls as the mechanism of injury.

In the second model, the reference categories were inverted (age 65‒79 years, moderate/high educational level grouped, and non-fall mechanisms of injury), allowing estimation of the odds ratios for the categories that served as reference groups in the first model.

It was found that TBI resulting from road traffic accidents was the most strongly correlated with a less favorable functional outcome, with an OR of 1.500 (95% CI: 1.040, 2.164) for GOSs 1‒4 and an OR of 2.865 (95% CI: 1.143, 7.181) for mortality. The other mechanisms presented no statistically significant risk for GOSs 1‒4; however, falls presented a lower risk of death than the other trauma mechanisms grouped together, with an OR of 0.592 (95% CI: 0.371, 0.945) (Table 5).

### Sensitivity analysis

Comparison between included and excluded patients in the sensitivity analysis showed no statistically significant differences in mean age (p = 0.27) or sex distribution (p = 0.41). However, a higher proportion of severe cases (GCS ≤ 8) was observed among excluded patients (15.1%vs. 9.1%; p = 0.048). In the extreme-case scenarios, the overall proportion of unfavorable outcomes ranged from 55.2% (favorable scenario) to 61.8% (unfavorable scenario), compared to 57.4% in the original model. The main associations ‒ age ≥ 80 years, low educational level, and presence of intracranial lesions ‒ retained similar magnitude and direction, with OR variation < 10% relative to the original model. In the multiple imputation analysis, adjusted estimates remained stable: OR for age ≥ 80 years = 1.41 (95% CI: 1.08–1.84) and OR for low educational level = 1.89 (95% CI: 1.22–2.92), comparable to the original model. Model AUC varied from 0.780 to 0.787, indicating that exclusions did not meaningfully impair predictive performance.

### The pandemic impact

Analyzing the progression of the pandemic, a slight tendency toward a decrease in monthly TBI cases was observed during months with higher regional COVID-19 incidence, although this correlation was not statistically significant (p = 0.325). The mean number of TBI cases per month during the study period was 11.2 (σ = 4.05), and only two cases were recorded in the two months following the first lockdown.

**Fig. 3:**
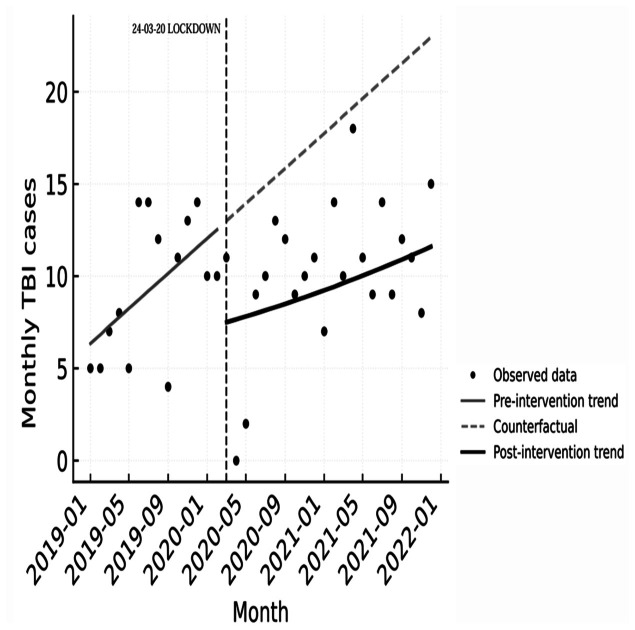
Standard Interrupted Time-Series (ITS) plot of monthly Traumatic Brain Injury (TBI) cases. Points show observed monthly counts (2019–2021). The vertical dashed line marks the intervention (first lockdown, March 2020). Note: State lockdown in accordance with Decree n° 64,881, OF March 22, 2020 was marked on the graph.

The Interrupted Time-Series (ITS) analysis revealed an immediate 42.3% reduction in monthly TBI incidence following the first lockdown (95% CI: −55.1% to −28.7%; p < 0.001), followed by a gradual increase of 2.1% per month (95% CI: 1.3%–2.9%; p < 0.001) until the end of 2021 (Fig. 3). Results were consistent across OLS-HAC and GLM Poisson models. Notably, the non-significant month-to-month association observed during the pandemic reflects within-period variability and should be distinguished from the abrupt level change identified by the ITS model.

The initial crude correlation between COVID-19 incidence and unfavorable TBI outcomes (*r* = 0.605) was substantially attenuated after adjustment for COVID-19 incidence, mobility restrictions, and total hospital admissions (adjusted *r* = 0.18; p = 0.09), suggesting confounding by factors such as reduced emergency care access and delayed presentation. Despite a proportional increase in severe cases during the pandemic, adjusted trends for mortality and unfavorable functional outcomes did not differ significantly from the pre-pandemic period (p = 0.11 and p = 0.08, respectively).

**Fig. 4:**
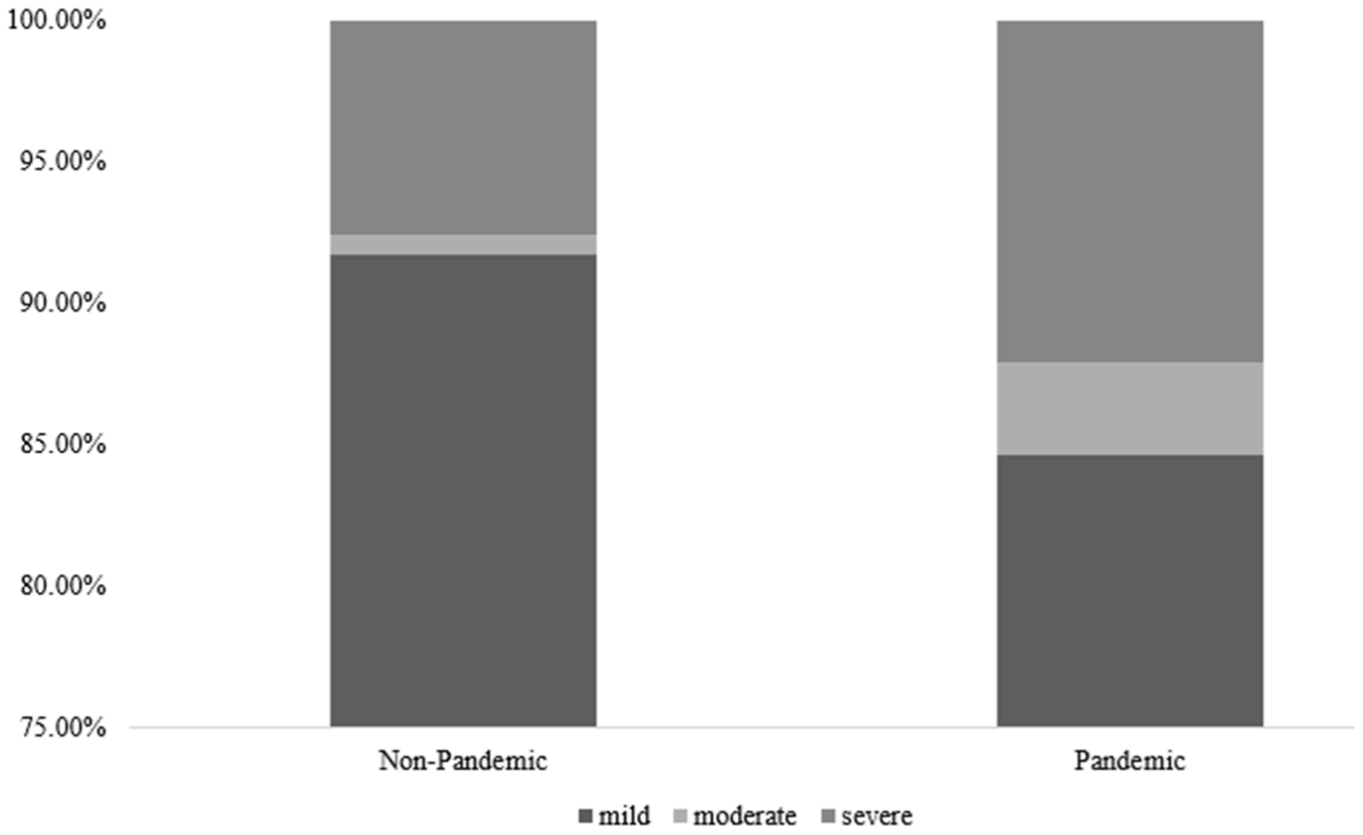
Comparative graph between the non-pandemic period (January 2010 to March 2020 and after May 2022) and pandemic period (March 2020 to May 2022), stratifying TBI cases into mild (GCS 13‒15), moderate (GCS 9‒12) and severe (GCS 3‒8) in percentages (p < 0.001; Chi-Square test for differences in proportions).

Comparing the 297 cases in the pandemic period (March 2020 to May 2022) and the 878 in non-pandemic period (January 2010 to March 2020 and after May 2022), a notable shift was observed in the profile of TBIs, with a decline in the proportion of cases classified as mild (from 91.7% to 84.6%) and an increase in those categorized as severe (from 7.6% to 12.1%) (Fig. 4).

**Fig. 5:**
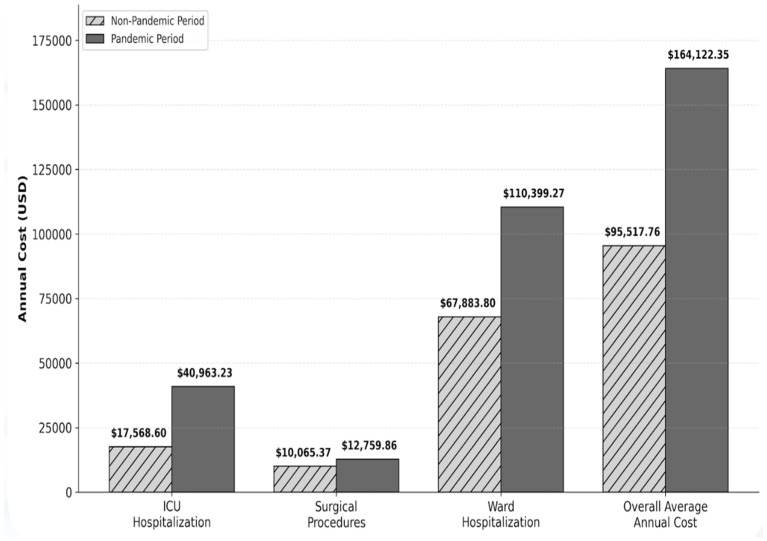
Comparison of average annual costs between pandemic and non-pandemic periods. Note: The graph illustrates the mean annual expenditures (in USD) for Intensive Care Unit (ICU) hospitalization, surgical procedures, and ward hospitalization, alongside the overall average annual cost. Light gray bars (with hatches) denote the non-pandemic period (2010–2020), while dark gray bars represent the pandemic period (2020–2022). All values reflect actual hospital expenditures, adjusted for inflation and converted to U.S. Dollars.

Annual costs associated with inpatient hospitalization increased by 62.63%, those associated with ICU hospitalization increased by 133.61%, and those associated with surgical intervention increased by 26.79%. Overall, there was a 71.82% increase in calculated annual direct costs and a 52.38% increase in the cost per patient associated with TBI ($1087.90 in the non-pandemic period to $1657.80 in the pandemic) (Fig. 5).

## Discussion

Elderly individuals are a vulnerable segment of the population and are susceptible to aging-related diseases. They are also at risk for injuries caused by external factors, such as trauma, specifically cranial trauma.^5^ There are many studies on TBI and the elderly population; however, few studies have specifically addressed Latin America and developing countries, especially in the southern hemisphere.

The present study revealed that falls were the most common cause of TBI in both periods. According to the literature, falls are responsible for more than one-third of all TBI cases in the general population and more than 60% of all TBI cases in people over 60-years of age.^6^^,^^7^ The incidence of falls is related to a mismatch between individual physiological functions, the environment, and behavioral factors. In older adults, falls can be caused by various risk factors, including advanced age, physical frailty, muscle weakness, unsteady gait, impaired balance, cognitive impairment, nutritional deficiencies, irregular sleep, visual impairment, chronic diseases, and overuse of medications, among others.^8^, ^9^, ^10^ Expanding access to evidence-based prevention measures, such as physical training to improve strength and balance, home safety evaluations, and regular medication reviews, is essential to reduce the incidence and impact of fall-related TBIs in elderly populations, particularly in low-resource settings. Randomized controlled trials and meta-analyses have consistently demonstrated the effectiveness of multifactorial interventions in preventing falls and related injuries in older adults.^11^^,^^12^ Unfortunately, falls are often not given the attention they deserve and are instead attributed to the aging process.^13^

This study revealed that age was a predictor of poor prognosis. Several factors contribute to the high mortality rates observed in elderly TBI patients. Understanding these factors is crucial for improving clinical outcomes and developing targeted interventions. Mortality rates increase significantly with age and the severity of the injury. For example, mortality begins to rise notably from age 56 and continues to increase with advancing age, regardless of the severity of the injury score. This age-related increase in mortality is likely due to diminished physiological reserves and the presence of comorbidities.^5^ Chronic health issues such as cardiovascular diseases, diabetes, and the use of anticoagulants and antiplatelet medications significantly increase the risk of complications and mortality in elderly TBI patients. These conditions complicate the management of TBI and can lead to poorer outcomes.^14^ Elderly patients generally experience worse functional outcomes than younger individuals do. They often suffer from higher rates of disability postinjury, and the likelihood of recovery diminishes with age. Additionally, complications such as respiratory failure, infections, and multiple organ failure are more prevalent in elderly individuals, further increasing mortality rates. For example, early positive cultures for infections significantly predict mortality in elderly TBI patients, underscoring the need for aggressive infection management.^15^ While surgical interventions can be beneficial for selected elderly patients, the overall outcomes are generally poorer than those of younger individuals. The decision for surgery must be carefully evaluated, considering factors such as the patient's level of consciousness, the radiologic type of injury, the mechanism of injury, and pupil abnormalities. Some elderly patients may derive little benefit from surgical intervention, and the risks may outweigh the potential benefits.^16^

While few population-based or multicenter studies have examined the distribution of TBI severity (mild, moderate, or severe) across different age groups, at least one nationally representative study in the U.S. reported a comparable distribution of TBI severity on the basis of the Glasgow Coma Scale (GCS).^17^ This finding aligns with the present study, where most TBIs were classified as mild.

With respect to sex distribution, epidemiological analyses from the USA and Europe have shown that the majority of the oldest patients with TBI are female and white.^17^, ^18^, ^19^ A Brazilian epidemiological study based on public health information revealed that the population over 80-years of age had the highest admission rate due to TBI, with approximately 138/100,000 year, followed by the 70–79 years of age group (92.5/100,000 year), with 20–29 years of follow-up (83/100,000 year).^20^ The present study revealed no relationship between sex, ethnicity, or occupation and functional outcome. Unfortunately, there is a lack of epidemiological studies on the epidemiology of TBI in developing countries.

Educational level may influence TBI outcomes through mechanisms related to health literacy, access to healthcare, socioeconomic conditions, and cognitive reserve. Although direct evidence specifically addressing educational attainment in elderly TBI populations remains limited, prior studies have reported associations between higher education and improved functional and neurocognitive outcomes in TBI cohorts. Asikainen et al. demonstrated the influence of age and educational level on social and vocational outcomes among TBI patients. They reported that patients with higher education levels generally had better outcomes, including greater functional independence and better vocational outcomes, even when they were injured early in life.^21^ Jung et al. investigated the prognostic factors for neurocognitive and functional outcomes in elderly TBI patients. They reported that educational level, along with age and sex, significantly influences neurocognitive outcomes, suggesting that higher education may offer some protective benefits or resilience against cognitive decline post-TBI.^22^ The present study revealed that in binary regression (excluding age), a low level of education was associated with poor functional outcomes. This finding is consistent with theories suggesting that education may serve as a marker of socioeconomic status, influencing exposure to injury risk, access to medical care, and living conditions. Additionally, individuals with lower schooling often demonstrate reduced health literacy, affecting their understanding of medical instructions, adherence to follow-up care, and engagement with rehabilitation services. A related mechanism is the concept of cognitive reserve, whereby higher education and intellectual enrichment build resilience against neurological injury, potentially mediating better outcomes after TBI.^23^^,^^24^ Moreover, early-life socioeconomic conditions are strongly associated with long-term cognitive outcomes following TBI, suggesting that educational level may also reflect broader social determinants shaping recovery trajectories.^25^

In addition to serving as a predictor, lower educational attainment likely reflects broader socioeconomic disadvantages, influencing health literacy, adherence to treatment, and participation in rehabilitation. To address these inequities, targeted policy responses are needed. Health literacy interventions ‒ such as simplified educational materials, structured discharge instructions, and caregiver training ‒ can improve comprehension and adherence to care. Robust evidence from a systematic review demonstrates that health literacy interventions among older adults significantly improve their ability to access, understand, and use health information.^26^ Furthermore, expanding rehabilitation and follow-up programs within the public health system, prioritizing vulnerable elderly groups with lower educational attainment, is essential. Integration of social support policies, such as community health worker outreach, may also help reduce disparities in access and outcomes.

Although the AUC values of 0.782 and 0.886 for unfavorable outcome and mortality, respectively, indicate strong discriminative ability, the Nagelkerke R^2^ values (0.245 and 0.401) suggest that a considerable portion of variance remains unexplained ‒ a common finding in complex biological outcomes. Rather than reflecting model inadequacy, this modest explained variance underscores the multifactorial nature of TBI prognosis in elderly patients. The models are therefore clinically useful for group-level risk stratification and identifying higher-risk subpopulations, even if not designed for precise individual prognostication. The absence of additional predictors such as pre-injury functional status, comorbidities, frailty indices, medication use, and cognitive status likely accounts for the residual variability and should be addressed in future prospective studies to enhance predictive accuracy and clinical applicability.

TBI in the elderly presents a significant financial burden on healthcare systems. The costs associated with TBI can vary on the basis of factors such as age, injury severity, and geographic location. Morris et al., using data from the Trauma Audit Research Network, investigated the costs of acute care in patients ≥18-years of age hospitalized for traumatic brain injury between January 2000 and December 2005 in England and Wales. The authors reported that hospitalization costs for TBI patients aged 18-years and above averaged £15,462. The costs varied significantly by age, injury severity, and presence of coexisting injuries. Older adults tend to have higher costs due to increased complications and longer hospital stays.^27^

A study from Maryland's Primary Adult Resource Center, USA, reported that for TBI patients aged 65-years and older, the mean unadjusted total hospitalization cost was $36,075. Physician charges accounted for 15% of total charges. The costs are lower for women than for men.^28^ In the Netherlands, the total annual cost of TBI was estimated at €314.6 million, with elderly individuals incurring higher direct healthcare costs. TBI in the elderly leads to significant economic and health burdens, emphasizing the need for targeted prevention and care strategies.^29^

The financial burden of TBI in the elderly population is substantial, with increasing costs over time and significant variability in terms of injury severity and geographic location. The costs in developing countries are underestimated and much lower than those in developed countries. In this study, the average cost per patient was $1152.11, ranging from $476.19 in 2010 to $1932.49 in 2021. These findings underscore the need for efficient healthcare policies and targeted prevention strategies to manage and mitigate the costs associated with TBI in elderly individuals. It is important to note, however, that this analysis of the true economic impact of TBI in this population is underestimated. Indirect costs, such as post-discharge rehabilitation, long-term care needs, and caregiver burden,^30^ were not included due to the cross-sectional and retrospective design of the study and the absence of imputable data in patient records.

Since the study took place during the pandemic, the authors cannot avoid discussing this peculiar period. The literature is abundant with studies on TBI and the elderly population; however, few studies have specifically addressed the epidemiological changes that occurred during the COVID-19 pandemic, with the systematic review and meta-analysis by Damara et al. standing out.^31^ This study, which involved 18,490 subjects from 13 different studies, demonstrated a significant decrease in hospital admissions for the elderly. This is likely due to the fact that some patients did not present with acute symptoms, and COVID-19 has a severe impact on elderly populations. Therefore, it is important for them to stay at home to prevent viral transmission. During the COVID-19 pandemic, a higher TBI mortality rate was observed in low-to-middle-income countries. Additionally, the proportion of subdural hemorrhage decreased while subarachnoid hemorrhage increased in low-to-middle-income and high-income countries, respectively. The proportion of TBI caused by assaults increased during the pandemic.

In terms of incidence, the present sample revealed a decrease in the average number of TBI cases during the pandemic period, especially during lockdown periods. This finding aligns with the observations made in the study conducted by Grassner et al. This encompassed data from all neurosurgical centers in the Czech Republic and the majority of Austria and Switzerland, representing a population of approximately 30-million individuals. The findings indicated a general downward trend in non-elective emergency neurosurgical cases during the COVID-19 pandemic.^32^

The interrupted time-series analysis demonstrated an immediate reduction in TBI admissions following the implementation of lockdown measures, reflecting a pronounced level change associated with mobility restrictions. In contrast, the absence of a statistically significant association between monthly COVID-19 incidence and TBI case numbers indicates that the observed decline was more closely related to structural mobility constraints than to fluctuations in viral transmission intensity. These findings reinforce the interpretation that the initial reduction in TBI admissions was primarily driven by abrupt societal restrictions, whereas subsequent variations in COVID-19 incidence did not independently influence TBI trends.

The Reid and Johnson study also examined the impact of the global COVID-19 pandemic on the hospital presentation of Traumatic Brain Injuries (TBIs), utilizing the Michigan Trauma Quality Improvement Program (MTQIP) database. The study found that the severity of TBI, as measured by the maximum AIS score for the head/neck region and the GCS score, remained unchanged throughout the pandemic.^33^ The present data indicate an increase in the number of cases classified as severe by the GCS during the pandemic, but mild and moderate cases still predominate. This finding is supported by the clinical management of these patients, with a significant increase in Intensive Care Unit (ICU) admissions and the need for neurosurgery.

In terms of functional outcomes, Petr et al. found no differences in the functional outcomes of TBI when comparing the non-pandemic period with the pandemic period in Central Europe.^34^ In contrast, Damara et al.’s meta-analysis indicated higher TBI mortality rates and a worse prognosis during the pandemic period in low- to middle-income countries.^31^

The COVID-19 pandemic has affected healthcare systems and providers worldwide in virtually all settings and specialties, presenting an unprecedented clinical and hospital management challenge. In this regard, this study showed a significant increase in average annual costs during the pandemic years, demonstrating rising costs and possibly losses, as expected by 84% of the trauma centers in Germany surveyed in the study by Schoeneberg et al.^35^ The 71.8% increase in direct hospital costs observed during the COVID-19 pandemic likely reflects a multifactorial phenomenon. Although the present study did not include qualitative interviews or direct protocol reviews, contextual factors described in the literature help interpret these findings. International reports have described disruptions in trauma care workflows, longer ICU stays due to infection control protocols, and increased complexity in managing trauma patients with concurrent COVID-19 infection.^36^ In addition, trauma patients who tested positive for COVID-19 experienced an average ICU length of stay nearly 3-days longer and hospital stays extended by approximately 2–3 days compared to COVID-negative trauma patients, after adjustment for age and injury severity.^37^ In the context of Brazil’s overstretched health system, prolonged stays and resource reallocation likely contributed significantly to higher per-patient costs during the pandemic.

The present study was inherently limited by the single-institution setting, which may limit generalizability of these findings to other settings, particularly in rural or less equipped hospitals. Single-center observational data are prone to local practice patterns and case-mix differences that may not reflect broader national or regional realities. Indeed, systematic reviews show that single-center trials often report larger effect sizes than multicenter studies, potentially overestimating associations.^38^ Nonetheless, single-center studies remain essential in settings where multicenter data are scarce, particularly in developing countries with limited research infrastructure. By providing detailed, context-specific insights, such studies help fill critical knowledge gaps and guide regional public health planning and resource allocation, especially where comparable data are not available. Furthermore, the present epidemiological findings ‒ such as the predominance of falls as the mechanism of injury, the increasing burden of TBI in elderly patients, and the associated cost profile ‒ are consistent with other Brazilian and Latin American single-center reports.^39^

The variable selection strategy adopted in this study was based on univariable screening (p < 0.05) to construct parsimonious multivariable models. Although this approach is acceptable in exploratory observational analyses, it may have excluded clinically relevant confounders that did not reach statistical significance in univariable testing, such as comorbidities or frailty, which represents a limitation of the present study. Consequently, residual confounding cannot be entirely excluded. Future studies should prioritize pre-specified multivariable models grounded in clinical and epidemiological rationale.

As a retrospective study, it relied on the accuracy of medical records and chart documentation, which introduces the possibility of coding and classification errors and restricts the ability to establish causal relationships between the observed variables. In addition, it was not possible to identify potential comorbidities prior to TBI. Additionally, because excluded patients had a higher proportion of severe TBI cases, their exclusion may have led to a slight underestimation of mortality and unfavorable functional outcomes. Finally, the present study did not explore the psychosocial impacts of Traumatic Brain Injury (TBI) on the elderly, which are crucial for a holistic understanding of patient recovery. Future research should incorporate these dimensions to provide a more comprehensive view of TBI consequences in this vulnerable population.

## Conclusion

This study presents a comprehensive epidemiological profile of Traumatic Brain Injury (TBI) in older adults treated at a tertiary trauma center in a developing country, highlighting the significant burden TBIs impose on this vulnerable population. Falls were identified as the predominant cause, with TBIs leading to increased hospital stays, higher healthcare costs, and substantial mortality rates, particularly among those with severe injuries and lower education levels.

The demographic analysis revealed that most TBI victims were over 80 years old, with no significant sex differences, and that lower education levels were correlated with poorer outcomes. The study also revealed a rising incidence of TBIs over the years, reflecting the growing elderly population and underscoring the need for enhanced trauma care and preventive strategies.

Overall, the findings emphasize the urgent need for targeted public health interventions to mitigate the burden of TBI among elderly populations in developing countries. Evidence-based fall prevention programs, including balance and strength training, home safety modifications, and regular medication review, should be implemented as a priority, given their proven effectiveness in reducing fall-related injuries in older adults. Additionally, the adoption of geriatric trauma protocols in emergency departments, encompassing standardized assessment pathways, early neuroimaging, careful anticoagulation management, and multidisciplinary evaluation, is crucial to improve outcomes. Finally, community-based awareness campaigns directed at older adults and their caregivers may play an important role in reinforcing the preventability of falls and ensuring timely access to care following trauma. Future research should continue to explore these trends and evaluate the effectiveness of targeted interventions to enhance the quality of life and healthcare outcomes for older adults with TBIs.

## Ethics approval

This study was approved by the Research Ethics Committee of the University Hospital Medical School of Ribeirão Preto (HCFMRP-USP), CAAE: 19261619.7.0000.5440. Number of the IRB: 3.532.220 Date of IRB approval: 26 August 2019. Due to the retrospective nature of the study, the requirement for informed consent was waived by the Research Ethics Committee.

## Declaration of generative AI in scientific writing

The authors declare that no generative Artificial Intelligence (AI) or AI-assisted technologies were used in the writing process of the manuscript.

## Data availability

The data sets generated and analyzed during the current study are not publicly available due to ethical and confidentiality restrictions but are available from the corresponding author on reasonable request.

## Authors’ contributions

Gabriel de Moraes Ribeiro: Conceptualization; methodology; formal analysis; investigation; data curation; writing-original draft, funding acquisition.

Matheus Ballestero: Conceptualization; methodology; investigation; data curation; writing-review & editing; supervision; project administration.

Leandro Candido de Souza: Methodology; software.

Lucas Rodrigues Olmedo: Data curation; writing-review & editing.

Rodrigo Inácio Pongeluppi: Software; investigation.

Benedicto Oscar Colli: Validation; resources; visualization; supervision.

Ricardo Santos de Oliveira: Conceptualization; methodology; validation; resources; writing-review & editing; visualization; supervision; project administration; funding acquisition.

## Declaration of competing interest

The authors report no conflict of interest concerning the materials or methods used in this study or the findings specified in this paper.

## Appendix Supplementary materials

Image, application 1

Image, application 2
