# Supplementary material for: Understanding traumatic brain injury in elderly populations: a developing country perspective
Source: Clinics (Sao Paulo). 2026 Jul 28;81:101054. doi: 10.1016/j.clinsp.2026.101054 (PMC13446349; doi:10.1016/j.clinsp.2026.101054)
Supplement: Supplementary file 1 [file mmc2.pdf]

HOSPITAL DAS CLÍNICAS DA FACULDADE DE MEDICINA
DE RIBEIRÃO PRETO DA UNIVERSIDADE DE SÃO PAULO
at-custos@hcrp.usp.br

FMRP-USP RIBEIRÃO PRETO

Custo Seção de
Assessoria Técnica

A Assessoria Técnica, através da Seção de Custos, divulga o custo médio de alguns serviços prestados no HCRP, durante os anos de 2008, 2009, 2010 e 2011.

Custo Médio dos Atendimentos

Unidades	Média Anual 2008	Média Anual 2009	Média Anual 2010	Média Anual 2011
Ambulatório	41,23	47,61	53,20	59,80
Vila Lobato [4]	52,41	58,73	63,27	- x -
UETDI	144,63	160,45	143,11	135,99
SAMSP + SESMT Medicina	60,01	58,98	63,61	55,55
CER - S. Médico Reabilitação	57,34	69,21	63,69	83,79
Casa 20	20,73	30,19	30,04	38,98
Sala de Urgência [1]	323,97	402,18	480,84	412,90
Pediatria - UE	392,67	475,91	503,70	438,50
Atendimento Queimados	354,54	382,16	222,38	172,52
Terapia Ocupacional	46,69	39,66	18,83	18,10
Cuidados Paliativos [2]	- x -	- x -	- x -	37,54
Psicologia - Campus [3]	37,68	37,90	41,47	- x -
Psicologia Psicodiagnóstico	- x -	- x -	- x -	42,42
Psicologia Clínica	- x -	- x -	- x -	78,46
Psicologia - UE	47,08	28,35	28,44	35,92
Serviço Social - Campus	28,12	34,10	35,52	37,84
Serviço Social - UE	34,05	47,04	48,30	53,57

Notas: (1) A Sala de Urgência engloba a Observação Adulto, Estabilização Clínica, G.O. Atendimento e Sala de Urgência.
(2) Cuidados Paliatvos foi criado no 4º Trimestre de 2010.
(3) A Psicologia Campus foi desmembrada em Psicologia Psicodiagnóstico e Psicologia Clínica a partir de 2011
(4) Vila Lobato está em reforma desde o final do ano de 2010

Custo Médio da Hora

	Média Anual 2008	Média Anual 2009	Média Anual 2010	Média Anual 2011
**Centro Cirúrgico**				
Central (sem Anestesia)	520,61	368,48	406,44	541,13
Anexo I (sem Anestesia)	390,87	531,72	523,18	602,55
Unid.de Emergência (com Anestesia)	613,24	543,62	661,34	795,69
Centro Cirúrgico Unidade de Queimados	833,84	938,07	997,07	1.064,90
**Anestesiologia**				
Campus	117,26	241,69	279,90	335,63
**Recuperação**				
Campus	36,14	33,23	32,96	40,61
Unidade de Emergência	21,90	21,33	28,13	29,08
**Centro Obstétrico**				
Parto	1.057,16	1.373,38	1.451,11	1.563,71
**Hemodiálise**				
Paciente crônico	140,00	136,00	154,00	138,00
Paciente agudo	210,00	204,00	231,00	207,00

Enfermarias (não são incluídos no custo do paciente-dia Exames Laboratoriais e de Diagnóstico)

TRANSPLANTE DE MEDULA OSSEA	Média Anual 2008	Média Anual 2009	Média Anual 2010	Média Anual 2011
Custo Médio do Leito-Dia	693,39	768,55	612,03	633,91
Custo Médio do Paciente-Dia	1.514,23	1.528,46	1.520,07	1.549,50
Custo Médio da Internação	13.022,38	16.660,21	17.328,80	19.833,60
**CLINICA MEDICA + DERMA**				
Custo Médio do Leito-Dia	197,69	215,14	211,80	216,42
Custo Médio do Paciente-Dia	419,72	434,01	447,08	462,99
Custo Médio da Internação	2.854,10	2.777,66	2.727,19	2.407,55
**UNIDADE METABOLICA**				
Custo Médio do Leito-Dia	224,71	263,18	249,94	288,63
Custo Médio do Paciente-Dia	381,11	482,59	500,64	657,90
Custo Médio da Internação	3.048,88	3.715,94	3.654,67	4.078,98
**MOLESTIAS INFECTOCONTAGIOSA**				
Custo Médio do Leito-Dia	211,87	234,59	215,28	230,90
Custo Médio do Paciente-Dia	334,84	416,50	381,21	413,43
Custo Médio da Internação	4.018,08	5.039,65	4.803,25	4.547,73
**CLINICA CIRURGICA CAMPUS**				
Custo Médio do Leito-Dia	155,45	172,04	172,87	191,51
Custo Médio do Paciente-Dia	338,31	365,76	398,54	437,75
Custo Médio da Internação	1.894,54	1.938,53	2.032,55	2.013,65
**UNIDADE DE NEUROCIRURGIA CAMPUS**				
Custo Médio do Leito-Dia	258,66	271,92	291,04	314,87
Custo Médio do Paciente-Dia	541,74	571,90	731,59	927,75
Custo Médio da Internação	2.058,61	2.058,84	2.414,25	3.432,68
**UNIDADE DE TRANSPLANTE DE FÍGADO**				
Custo Médio do Leito-Dia	458,02	526,03	517,12	546,49
Custo Médio do Paciente-Dia	869,66	913,05	1.000,03	1.001,50
Custo Médio da Internação	5.217,96	5.569,61	4.800,14	4.907,35
**ENFERMARIA DE PSIQUIATRIA**				
Custo Médio do Leito-Dia	275,92	305,57	311,59	297,80
Custo Médio do Paciente-Dia	409,91	450,72	460,66	509,17
Custo Médio da Internação	4.181,08	4.462,13	4.284,14	3.920,61
**ENFERMARIA DE NEUROLOGIA**				
Custo Médio do Leito-Dia	143,12	173,28	160,14	179,46
Custo Médio do Paciente-Dia	384,34	571,39	460,30	499,71
Custo Médio da Internação	1.652,66	2.171,28	1.795,17	2.248,70
**ENFERMARIA DE ORTOPEDIA**				
Custo Médio do Leito-Dia	129,40	144,26	150,40	154,76
Custo Médio do Paciente-Dia	268,72	306,45	323,94	355,13
Custo Médio da Internação	1.639,19	1.746,77	2.008,43	2.770,01

ENFERMARIA DE OFTALMOLOGIA

Custo Médio do Leito-Dia	73,24	80,62	76,63	- x -
Custo Médio do Paciente-Dia	216,80	199,09	203,30	- x -
Custo Médio da Internação	650,40	637,09	711,55	- x -

ENFERMARIA DE OTORRINOLARINGOLOGIA

Custo Médio do Leito-Dia	111,79	114,90	104,70	- x -
Custo Médio do Paciente-Dia	292,03	299,65	291,05	- x -
Custo Médio da Internação	642,47	719,16	727,63	- x -

ENFERMARIA DE CIRURGIA DE CABEÇA E PESCOÇO

Custo Médio do Leito-Dia	238,31	256,14	259,16	- x -
Custo Médio do Paciente-Dia	454,69	477,64	471,13	- x -
Custo Médio da Internação	1.773,29	1.719,50	1.978,75	- x -

ENFERMARIA 12º ANDAR-OFT-ORL-CCP

Custo Médio do Leito-Dia	- x -	- x -	- x -	140,64
Custo Médio do Paciente-Dia	- x -	- x -	- x -	372,01
Custo Médio da Internação	- x -	- x -	- x -	1.450,84

A partir de 2011 foi unificado o 12º andar OFT + OTR e CCP

ENFERMARIA DE GINECOLOGIA E OBSTETRÍCIA

Custo Médio do Leito-Dia	135,19	149,97	151,31	153,11
Custo Médio do Paciente-Dia	262,66	298,89	296,06	334,79
Custo Médio da Internação	971,84	1.135,78	1.184,24	1.272,20

ENFERMARIA DE PEDIATRIA CAMPUS

Custo Médio do Leito-Dia	246,36	274,08	280,53	303,07
Custo Médio do Paciente-Dia	555,62	602,00	626,56	712,97
Custo Médio da Internação	3.167,03	3.732,40	3.571,39	4.563,01

C.T.I. PEDIÁTRICO CAMPUS

Custo Médio do Leito-Dia	695,51	729,87	749,49	851,03
Custo Médio do Paciente-Dia	1.408,03	1.445,98	1.424,00	1.784,35
Custo Médio da Internação	9.574,60	10.700,25	10.964,80	11.241,41

UNIDADE POS OPERATÓRIO DO TORAX

Custo Médio do Leito-Dia	448,99	522,07	489,71	521,94
Custo Médio do Paciente-Dia	1.244,79	1.294,37	1.176,09	1.860,23
Custo Médio da Internação	1.991,66	2.977,05	3.175,44	3.906,48

UETDI - ENFERMARIA

Custo Médio do Leito-Dia	306,23	347,38	355,34	378,15
Custo Médio do Paciente-Dia	533,67	604,96	639,92	680,26
Custo Médio da Internação	7.418,01	10.647,30	8.382,95	7.482,86

C.T.I. ADULTO CAMPUS

Custo Médio do Leito-Dia	903,58	1.019,01	1.081,93	1.174,63
Custo Médio do Paciente-Dia	1.413,95	1.670,79	1.872,15	1.924,52
Custo Médio da Internação	13.432,53	13.533,40	14.228,34	12.316,93

UNIDADE CORONARIANA

Custo Médio do Leito-Dia	525,61	584,71	638,77	646,37
Custo Médio do Paciente-Dia	845,46	821,93	908,13	925,98
Custo Médio da Internação	4.396,39	3.123,33	3.995,77	4.815,10

UTR (2º ANDAR)

Custo Médio do Leito-Dia	384,95	423,59	298,14	308,07
Custo Médio do Paciente-Dia	611,83	647,28	598,99	568,83
Custo Médio da Internação	3.059,15	3.948,41	2.875,15	2.787,27

BERÇÁRIO 7º C

Custo Médio do Leito-Dia	437,90	473,66	479,39	498,58
Custo Médio do Paciente-Dia	783,10	805,39	844,87	903,60
Custo Médio da Internação	10.101,99	10.228,45	11.405,75	11.114,28

BERÇARIO 8º C

Custo Médio do Leito-Dia	232,95	255,83	278,25	287,92
Custo Médio do Paciente-Dia	377,92	419,95	452,95	495,14
Custo Médio da Internação	1.360,51	1.679,80	1.947,69	1.980,56

CIREP

Custo Médio do Leito-Dia	891,96	1.057,69	1.137,38	1.172,85
Custo Médio do Paciente-Dia	1.571,32	1.969,93	2.040,47	2.206,01
Custo Médio da Internação	7.856,60	10.637,62	9.386,16	9.044,64

UNIDADE DE TERAPIA IMUNOLOGICA

Custo Médio do Leito-Dia	518,19	493,90	596,71	640,41
Custo Médio do Paciente-Dia	1.475,08	1.072,73	1.323,80	1.276,50
Custo Médio da Internação	5.457,80	4.076,37	4.500,92	5.616,60

ENFERMARIA DE ONCOLOGIA

Custo Médio do Leito-Dia	- x -	- x -	- x -	302,78
Custo Médio do Paciente-Dia	- x -	- x -	- x -	973,74
Custo Médio da Internação	- x -	- x -	- x -	4.381,82

Criada a partir do 2º Semestre de 2011.

ENFERMARIA DE CLÍNICA CIRURGIA - UE (Inclui: Cir.Urg., Ortop., C.C.P., Gineco., Semi Intens., Isolamento)

Custo Médio do Leito-Dia	209,15	245,55	253,30	262,62
Custo Médio do Paciente-Dia	278,78	315,46	313,91	402,83
Custo Médio da Internação	947,86	1.072,58	1.192,86	1.571,04

ENFERMARIA DE NEUROCLINICA

Custo Médio do Leito-Dia	285,77	357,27	- x -	- x -
Custo Médio do Paciente-Dia	291,52	351,47	- x -	- x -
Custo Médio da Internação	1.107,79	1.616,74	- x -	- x -

* A partir do 3º trimestre de 2009 essa Unidade foi desmembrada entre Cl. Médica e Neurologia.

ENFERMARIA DE NEUROCLÍNICA - SEMI INTENSIVO

Custo Médio do Leito-Dia	304,41	375,82	- x -	- x -
Custo Médio do Paciente-Dia	468,58	559,48	- x -	- x -
Custo Médio da Internação	4.264,06	5.035,36	- x -	- x -

* A partir do 3º trimestre de 2009 essa Unidade foi desmembrada entre Cl. Médica e Neurologia.

ENFERMARIA DE NEUROCLÍNICA - ISOLAMENTO

Custo Médio do Leito-Dia	300,60	359,41	- x -	- x -
Custo Médio do Paciente-Dia	461,73	548,11	- x -	- x -
Custo Médio da Internação	3.047,44	2.904,96	- x -	- x -

* A partir do 3º trimestre de 2009 essa Unidade foi desmembrada entre Cl. Médica e Neurologia.

ENFERMARIA DE CLÍNICA MÉDICA - UE

Custo Médio do Leito-Dia	- x -	282,14	396,99	353,37
Custo Médio do Paciente-Dia	- x -	382,51	423,35	548,69
Custo Médio da Internação	- x -	1.683,03	1.651,05	4.334,68

* Foi desmembrada da Neuroclínica a partir do 3º Trim/2009

ENFERMARIA NEUROLOGIA - UE

Custo Médio do Leito-Dia	- x -	320,53	361,53	327,16
Custo Médio do Paciente-Dia	- x -	385,08	399,01	485,03
Custo Médio da Internação	- x -	1.886,87	2.394,06	4.753,27

* Foi desmembrada da Neuroclínica a partir do 3º Trim/2009

UNIDADE CORONARIANA - UE

Custo Médio do Leito-Dia	- x -	605,50	700,31	792,18
Custo Médio do Paciente-Dia	- x -	1.743,65	1.218,61	1.198,92
Custo Médio da Internação	- x -	5.579,68	4.752,60	4.436,00

* Criada a partir do 3º trimestre de 2009.

ENFERMARIA DE PEDIATRIA U.E.

Custo Médio do Leito-Dia	255,90	301,88	328,08	335,43
Custo Médio do Paciente-Dia	431,75	478,95	526,30	575,36
Custo Médio da Internação	2.158,76	2.346,84	2.631,48	4.027,50

ENFERMARIA DE MI INFANTIL U.E.

Custo Médio do Leito-Dia	286,78	312,37	288,82	293,41
Custo Médio do Paciente-Dia	498,18	574,44	491,54	478,46
Custo Médio da Internação	3.686,53	3.906,19	3.686,57	4.354,01

ENFERMARIA DE UNID.DE QUEIMADOS

Custo Médio do Leito-Dia	290,47	319,23	369,06	380,26
Custo Médio do Paciente-Dia	481,88	481,06	556,78	560,93
Custo Médio da Internação	5.589,79	6.157,55	7.293,88	8.582,25

C.T.I. ADULTO - U.E.

Custo Médio do Leito-Dia	658,74	770,26	883,53	1.055,17
Custo Médio do Paciente-Dia	1.002,55	1.146,42	1.325,47	1.575,61
Custo Médio da Internação	9.423,98	11.578,85	13.917,41	16.701,50

C.T.I. PEDIÁTRICO - U.E.

Custo Médio do Leito-Dia	889,38	718,29	756,27	858,93
Custo Médio do Paciente-Dia	1.397,15	1.388,72	1.442,76	1.608,60
Custo Médio da Internação	10.478,64	11.804,11	11.109,21	14.638,24

Gislaine R. Guimarães
Responsável pela Seção de Custos

A Assessoria Técnica, através da Seção de Custos, divulga o custo médio de alguns serviços prestados no HCRP, durante os anos de 2012, 2013 e 2014.

Custo Médio Atendimentos

Unidades	Média Anual 2012	Média Anual 2013	Média Anual 2014
Ambulatório	63,39	77,02	81,01
UETDI	143,36	156,76	184,98
CER - S. Médico Reabilitação	67,47	97,65	128,82
Casa 20	45,37	50,45	46,42
Sala de Urgência [1]	430,21	454,28	606,65
Pediatria - UE	378,44	445,07	583,58
Atendimento Queimados	133,77	155,65	169,14
Terapia Ocupacional	20,87	18,53	25,24
Cuidados Paliativos	39,47	45,48	57,60
Psicologia Psicodiagnóstico	37,94	39,02	48,57
Psicologia Clínica	102,32	91,68	101,87
Psicologia - UE	26,39	25,27	27,97

Notas: (1) A Sala de Urgência engloba a Observação Adulto, Estabilização Clínica, G.O. Atendimento e Sala de Urgência.

Custo Médio da Hora

	Média Anual 2012	Média Anual 2013	Média Anual 2014
**Centro Cirúrgico**			
Central (sem Anestesia)	501,85	625,31	690,88
Anexo I (sem Anestesia) [1]	- x -	- x -	- x -
Unid.de Emergência (com Anestesia)	809,98	514,01	673,78
Centro Cirúrgico Unidade de Queimados	884,02	1.151,68	953,56

Notas: (1) Área desativada a partir de julho/2012

	Média Anual 2012	Média Anual 2013	Média Anual 2014
**Anestesiologia**			
Campus	300,15	388,40	454,06
**Recuperação**			
Campus	37,37	42,68	40,26
Unidade de Emergência	32,32	39,19	39,97
**Centro Obstétrico**			
Parto	1.720,89	2.276,58	2.846,72
**Hemodiálise**			
Paciente crônico	194,00	204,00	228,00
Paciente agudo	291,00	306,00	342,00

Enfermarias (não são incluídos no custo do paciente-dia Exames Laboratoriais e de Diagnóstico)

TRANSPLANTE MEDULA OSSEA	Média Anual 2012	Média Anual 2013	Média Anual 2014
Custo Médio do Leito-Dia	662,67	669,24	778,77
Custo Médio do Paciente-Dia	1.881,92	1.705,99	2.042,84
Custo Médio da Internação	29.357,95	23.883,86	26.761,20

CLINICA MÉDICA 5º e 6º andares [1]			
Custo Médio do Leito-Dia	238,64	268,21	278,68
Custo Médio do Paciente-Dia	530,97	564,04	576,45
Custo Médio da Internação	2.761,04	3.102,22	3.170,48

Notas: (1) A partir de 2012 somado a Unidade Metabólica e desmembrado a Derma e Imuno

(1) A partir do 2º trim/2014 desmembrado a Enfermaria Hemato-Químio

CLINICA MÉDICA 4º andar (Derma e Imuno)			
Custo Médio do Leito-Dia	243,90	255,67	266,48
Custo Médio do Paciente-Dia	555,96	620,87	652,41
Custo Médio da Internação	3.002,18	2.856,00	2.805,36

Notas: (1) Desmembrado da Cl. Médica a partir de 2012

MOLESTIAS INFECTOCONTAGIOSA			
Custo Médio do Leito-Dia	256,92	289,24	318,33
Custo Médio do Paciente-Dia	475,33	552,29	614,74
Custo Médio da Internação	5.894,09	5.964,73	6.639,19

CLINICA CIRURGICA CAMPUS [1]			
Custo Médio do Leito-Dia	211,53	232,81	302,45
Custo Médio do Paciente-Dia	443,16	472,00	593,39
Custo Médio da Internação	2.038,54	2.124,00	2.610,92

Notas: (1) Somado a Neurocirurgia a partir do 4º trim/2014

UNIDADE DE NEUROCIRURGIA CAMPUS [1]			
Custo Médio do Leito-Dia	313,09	359,75	418,55
Custo Médio do Paciente-Dia	826,79	831,98	828,42
Custo Médio da Internação	3.059,12	2.745,53	2.733,79

Notas: (1) Somado no grupo da Cl Cirúrgica a partir do 4º trim/2014

UNIDADE DE TRANSPLANTE DE FÍGADO			
Custo Médio do Leito-Dia	611,89	780,41	723,32
Custo Médio do Paciente-Dia	1.166,35	1.343,07	1.202,25
Custo Médio da Internação	5.481,85	7.789,81	7.333,73

ENFERMARIA DE PSIQUIATRIA			
Custo Médio do Leito-Dia	330,48	394,42	410,33
Custo Médio do Paciente-Dia	518,13	562,86	602,21
Custo Médio da Internação	4.093,23	5.797,46	6.262,98

ENFERMARIA DE NEUROLOGIA			
Custo Médio do Leito-Dia	192,48	227,16	277,96
Custo Médio do Paciente-Dia	467,67	510,94	576,16
Custo Médio da Internação	2.198,05	2.554,70	3.341,73

ENFERMARIA DE ORTOPEDIA

Custo Médio do Leito-Dia	174,24	202,31	216,97
Custo Médio do Paciente-Dia	370,76	410,80	476,09
Custo Médio da Internação	2.484,09	2.752,36	2.618,50

ENFERMARIA 12º ANDAR (OFT-ORL-CCP)

Custo Médio do Leito-Dia	152,21	170,93	248,86
Custo Médio do Paciente-Dia	369,16	425,01	596,05
Custo Médio da Internação	1.255,14	1.275,03	1.788,15

ENFERMARIA DE GINECOLOGIA E OBSTETRÍCIA

Custo Médio do Leito-Dia	168,94	183,20	194,39
Custo Médio do Paciente-Dia	360,04	401,21	377,49
Custo Médio da Internação	1.512,17	1.644,96	1.774,20

ENFERMARIA DE PEDIATRIA CAMPUS

Custo Médio do Leito-Dia	323,09	378,84	431,26
Custo Médio do Paciente-Dia	784,15	981,13	1.072,42
Custo Médio da Internação	4.156,00	5.199,99	5.040,37

C.T.I. PEDIÁTRICO CAMPUS

Custo Médio do Leito-Dia	971,95	1.143,57	1.138,59
Custo Médio do Paciente-Dia	1.740,49	2.011,17	1.969,77
Custo Médio da Internação	15.142,26	15.687,13	17.333,98

UNIDADE POS OPERATÓRIO DO TORAX [1]

Custo Médio do Leito-Dia	519,86	584,70	684,02
Custo Médio do Paciente-Dia	1.193,08	1.245,54	1.457,33
Custo Médio da Internação	2.863,39	2.740,19	3.351,86

Notas: (1) Área desativada a partir do 3º trim/2014

UETDI - ENFERMARIA

Custo Médio do Leito-Dia	493,51	565,34	630,34
Custo Médio do Paciente-Dia	725,11	805,01	899,26
Custo Médio da Internação	11.094,18	11.592,14	13.758,68

C.T.I. ADULTO CAMPUS

Custo Médio do Leito-Dia	1.332,80	1.565,45	1.773,72
Custo Médio do Paciente-Dia	2.135,19	2.413,46	2.877,40
Custo Médio da Internação	16.654,48	14.963,45	18.990,84

UNIDADE CORONARIANA

Custo Médio do Leito-Dia	591,45	633,54	767,81
Custo Médio do Paciente-Dia	873,20	932,91	1.039,88
Custo Médio da Internação	5.326,52	5.784,04	4.991,42

UTR (2º ANDAR)

Custo Médio do Leito-Dia	355,38	453,99	542,82
Custo Médio do Paciente-Dia	634,43	679,54	803,44
Custo Médio da Internação	3.045,26	3.397,70	5.302,70

BERÇÁRIO 7º C

Custo Médio do Leito-Dia	577,24	773,26	864,41
Custo Médio do Paciente-Dia	1.008,56	1.226,10	1.251,58
Custo Médio da Internação	10.186,46	15.081,03	16.020,22

BERÇARIO 8º C

Custo Médio do Leito-Dia	299,86	352,39	398,45
Custo Médio do Paciente-Dia	497,88	598,65	648,67
Custo Médio da Internação	1.891,94	2.394,60	2.724,41

CIREP

Custo Médio do Leito-Dia	1.234,78	1.363,40	2.191,64
Custo Médio do Paciente-Dia	1.828,81	2.083,01	2.504,30
Custo Médio da Internação	8.046,76	8.748,64	10.768,49

UNIDADE DE TERAPIA IMUNOLOGICA

Custo Médio do Leito-Dia	712,38	832,18	955,76
Custo Médio do Paciente-Dia	1.560,11	1.772,39	2.015,55
Custo Médio da Internação	5.304,37	7.089,56	9.069,98

ENFERMARIA DE ONCOLOGIA

Custo Médio do Leito-Dia	366,11	468,50	485,79
Custo Médio do Paciente-Dia	888,57	936,68	941,94
Custo Médio da Internação	4.265,16	4.027,73	4.238,71

ENFERMARIA HEMATO-QUÍMIO 6º ANDAR [1]

Custo Médio do Leito-Dia	- x -	- x -	545,37
Custo Médio do Paciente-Dia	- x -	- x -	1.471,77
Custo Médio da Internação	- x -	- x -	11.921,31

Notas: (1) Desmembrado da Cl. Médica a partir do 2º trim/2014

UNIDADE TERAPIA INTENSIVA PÓS-OPERATÓRIA

Custo Médio do Leito-Dia	- x -	- x -	602,40
Custo Médio do Paciente-Dia	- x -	- x -	2.033,43
Custo Médio da Internação	- x -	- x -	4.066,85

Criado a partir do 3º trim/2014

ENFERMARIA DE CLÍNICA CIRURGIA - UE (Inclui: Cir.Urg., Ortop., C.C.P., Gineco., Semi Intens., Isolar

Custo Médio do Leito-Dia	304,40	364,04	447,17
Custo Médio do Paciente-Dia	452,73	520,27	637,68
Custo Médio da Internação	1.946,73	2.081,09	2.423,17

ENFERMARIA DE CLÍNICA MÉDICA - UE

Custo Médio do Leito-Dia	446,61	530,59	605,97
Custo Médio do Paciente-Dia	692,73	765,24	863,31
Custo Médio da Internação	4.017,81	5.280,15	5.611,52

ENFERMARIA NEUROLOGIA - UE

Custo Médio do Leito-Dia	364,78	419,03	490,21
Custo Médio do Paciente-Dia	546,31	582,63	699,37
Custo Médio da Internação	6.774,28	8.273,31	6.574,06

**UNIDADE CORONARIANA - UE**			
Custo Médio do Leito-Dia	1.040,30	1.294,24	1.347,22
Custo Médio do Paciente-Dia	1.342,54	1.573,14	1.743,29
Custo Médio da Internação	6.041,44	8.809,59	8.542,10
**ENFERMARIA DE PEDIATRIA U.E.**			
Custo Médio do Leito-Dia	360,25	441,02	513,65
Custo Médio do Paciente-Dia	657,30	733,48	935,75
Custo Médio da Internação	4.206,71	4.620,94	4.959,50
**ENFERMARIA DE MI INFANTIL U.E.**			
Custo Médio do Leito-Dia	362,99	424,19	438,41
Custo Médio do Paciente-Dia	620,14	676,70	798,53
Custo Médio da Internação	5.209,14	5.345,92	6.627,82
**ENFERMARIA DE UNID.DE QUEIMADOS**			
Custo Médio do Leito-Dia	431,41	488,19	523,58
Custo Médio do Paciente-Dia	605,96	658,58	784,65
Custo Médio da Internação	9.816,49	12.513,01	14.359,10
**C.T.I. ADULTO - U.E.**			
Custo Médio do Leito-Dia	1.205,38	1.496,22	1.547,98
Custo Médio do Paciente-Dia	1.723,76	1.938,35	2.039,30
Custo Médio da Internação	20.857,51	25.198,57	24.063,72
**C.T.I. PEDIÁTRICO - U.E.**			
Custo Médio do Leito-Dia	918,53	1.124,63	1.318,69
Custo Médio do Paciente-Dia	1.655,51	1.682,09	2.276,83
Custo Médio da Internação	13.078,49	16.148,08	21.857,55

Seção de Custos, 14 de abril de 2015

A Assessoria Técnica, através da Seção de Custos, divulga o custo médio de alguns serviços prestados no HCRP, durante os anos de 2015 a 2017.

Custo Médio Atendimentos

	Média Anual 2015	Média Anual 2016	Média Anual 2017
Ambulatório	79,71	71,80	75,78
Ambulatório HC CRIANÇA	142,03	110,96	117,99
UETDI	199,24	204,41	201,85
CER - S. Médico Reabilitação	109,15	95,87	102,25
Casa 20	59,88	63,44	- x -
Sala de Urgência [1]	624,71	596,02	610,06
Pediatria - UE	608,54	510,90	558,29
Atendimento Queimados	148,19	126,36	136,20

Notas: (1) A Sala de Urgência engloba a Observação Adulto, Estabilização Clínica, G.O. Atendimento e Sala de Urgência.

Custo Médio da Hora

	Média Anual 2015	Média Anual 2016	Média Anual 2017
**Centro Cirúrgico**			
Central (sem Anestesia)	692,30	684,57	688,45
Unid.de Emergência (com Anestesia)	824,00	804,78	814,60
**Anestesiologia**			
Campus	430,53	430,05	430,29
**Recuperação**			
Campus	36,11	39,38	37,65
Unidade de Emergência	38,84	53,48	45,50
**Hemodiálise**			
Paciente crônico	216,00	266,00	242,00
Paciente agudo	324,00	399,00	363,00

Enfermarias (não são incluídos no custo do paciente-dia Exames Laboratoriais e de Diagnóstico)

TRANSPLANTE MEDULA OSSEA

	Média Anual 2015	Média Anual 2016	Média Anual 2017
Custo Médio do Leito-Dia	709,20	775,13	741,86
Custo Médio do Paciente-Dia	1.636,63	1.942,36	1.784,26
Custo Médio da Internação	25.367,77	30.883,52	28.012,88

CLINICA MÉDICA 5º e 6º andares [1]

	Média Anual 2015	Média Anual 2016	Média Anual 2017
Custo Médio do Leito-Dia	269,41	256,87	262,87
Custo Médio do Paciente-Dia	544,61	516,88	530,39
Custo Médio da Internação	3.322,12	2.946,22	3.129,30

Notas: (1) A partir de 2012 somado a Unidade Metabólica e desmembrado a Derma e Imuno

(1) A partir do 2º trim/2014 desmembrado a Enfermaria Hemato-Químio

CLINICA MÉDICA 4º andar (Derma e Imuno)

	Média Anual 2015	Média Anual 2016	Média Anual 2017
Custo Médio do Leito-Dia	285,88	256,48	270,74
Custo Médio do Paciente-Dia	692,87	583,74	636,87
Custo Médio da Internação	2.702,19	2.685,20	2.706,70

Notas: (1) Desmembrado da Cl. Médica a partir de 2012

	Média Anual 2015	Média Anual 2016	Média Anual 2017
**MOLESTIAS INFECTOCONTAGIOSA**			
Custo Médio do Leito-Dia	314,26	315,84	314,83
Custo Médio do Paciente-Dia	718,84	637,14	677,86
Custo Médio da Internação	8.051,01	6.435,11	7.219,21
**CLINICA CIRURGICA CAMPUS [1]**			
Custo Médio do Leito-Dia	327,92	281,36	303,61
Custo Médio do Paciente-Dia	659,01	590,79	622,87
Custo Médio da Internação	2.701,94	2.540,40	2.616,05

Notas: (1) Somado a Neurocirurgia a partir do 4º trim/2014

	Média Anual 2015	Média Anual 2016	Média Anual 2017
**UNIDADE DE TRANSPLANTE DE FÍGADO**			
Custo Médio do Leito-Dia	855,51	818,32	834,79
Custo Médio do Paciente-Dia	1.388,33	1.391,75	1.389,90
Custo Médio da Internação	5.553,32	6.958,75	6.254,55
**ENFERMARIA DE PSIQUIATRIA**			
Custo Médio do Leito-Dia	433,88	400,85	417,23
Custo Médio do Paciente-Dia	655,78	582,46	618,47
Custo Médio da Internação	5.902,02	5.824,60	5.875,47
**ENFERMARIA DE NEUROLOGIA**			
Custo Médio do Leito-Dia	295,58	279,34	287,14
Custo Médio do Paciente-Dia	605,77	558,76	581,57
Custo Médio da Internação	3.392,31	3.240,81	3.314,95
**ENFERMARIA DE ORTOPEDIA**			
Custo Médio do Leito-Dia	216,82	215,11	215,73
Custo Médio do Paciente-Dia	481,01	507,48	492,96
Custo Médio da Internação	2.741,76	2.892,64	2.809,87
**ENFERMARIA 12º ANDAR (OFT-ORL-CCP)**			
Custo Médio do Leito-Dia	242,10	201,07	219,47
Custo Médio do Paciente-Dia	587,15	575,94	581,08
Custo Médio da Internação	2.113,74	1.900,60	2.004,73
**ENFERMARIA DE GINECOLOGIA E OBSTETRÍCIA**			
Custo Médio do Leito-Dia	196,77	209,72	202,98
Custo Médio do Paciente-Dia	407,74	413,05	410,12
Custo Médio da Internação	1.794,06	1.941,34	1.866,05
**ENFERMARIA DE PEDIATRIA CAMPUS**			
Custo Médio do Leito-Dia	453,86	443,96	448,48
Custo Médio do Paciente-Dia	929,47	990,42	958,56
Custo Médio da Internação	5.390,93	5.249,23	5.320,01
**C.T.I. PEDIÁTRICO CAMPUS**			
Custo Médio do Leito-Dia	1.216,32	1.127,47	1.171,42
Custo Médio do Paciente-Dia	1.978,24	1.857,23	1.918,36
Custo Médio da Internação	20.771,52	13.372,06	16.977,49
**UNIDADE POS OPERATÓRIO DO TORAX [1]**			
Custo Médio do Leito-Dia	739,39	747,01	743,02
Custo Médio do Paciente-Dia	1.612,48	1.841,25	1.723,31
Custo Médio da Internação	4.192,44	5.339,62	4.739,10

Notas: (1) Área desativada a partir do 3º trim/2014

	Média Anual 2015	Média Anual 2016	Média Anual 2017
**UETDI - ENFERMARIA**			
Custo Médio do Leito-Dia	630,90	569,09	599,75
Custo Médio do Paciente-Dia	938,28	837,25	887,59
Custo Médio da Internação	12.760,61	10.381,90	11.538,67
**C.T.I. ADULTO CAMPUS**			
Custo Médio do Leito-Dia	1.785,01	1.688,28	1.736,52
Custo Médio do Paciente-Dia	2.686,61	2.529,94	2.607,10
Custo Médio da Internação	19.343,59	20.998,50	20.205,03
**UNIDADE CORONARIANA**			
Custo Médio do Leito-Dia	1.211,40	1.283,73	1.247,06
Custo Médio do Paciente-Dia	1.549,33	1.694,70	1.621,48
Custo Médio da Internação	7.436,78	11.523,96	9.404,58
**UTR** (2º ANDAR)			
Custo Médio do Leito-Dia	553,45	503,38	528,03
Custo Médio do Paciente-Dia	969,34	873,75	922,16
Custo Médio da Internação	5.234,44	5.941,50	5.625,18
**BERÇÁRIO 7º C**			
Custo Médio do Leito-Dia	899,95	857,53	878,47
Custo Médio do Paciente-Dia	1.241,08	1.200,64	1.220,86
Custo Médio da Internação	16.134,04	14.887,94	15.504,92
**BERÇARIO 8º C**			
Custo Médio do Leito-Dia	387,49	379,10	383,09
Custo Médio do Paciente-Dia	608,67	616,18	612,14
Custo Médio da Internação	2.556,41	2.587,96	2.570,99
**CIREP**			
Custo Médio do Leito-Dia	2.363,09	2.248,42	2.305,46
Custo Médio do Paciente-Dia	2.643,26	2.527,90	2.585,40
Custo Médio da Internação	10.308,71	10.111,60	10.212,33
**UNIDADE DE TERAPIA IMUNOLOGICA**			
Custo Médio do Leito-Dia	930,40	1.002,83	963,82
Custo Médio do Paciente-Dia	2.006,98	1.702,29	1.851,61
Custo Médio da Internação	7.425,83	7.319,85	7.406,44
**ENFERMARIA DE ONCOLOGIA**			
Custo Médio do Leito-Dia	468,79	436,52	452,43
Custo Médio do Paciente-Dia	868,80	794,00	830,23
Custo Médio da Internação	3.735,83	3.414,21	3.569,99
**ENFERMARIA HEMATO-QUÍMIO 6º ANDAR** [1]			
Custo Médio do Leito-Dia	536,65	494,02	514,85
Custo Médio do Paciente-Dia	1.501,46	1.343,55	1.423,99
Custo Médio da Internação	10.360,07	9.673,57	10.039,13

Notas: (1) Desmembrado da Cl. Médica a partir do 2º trim/2014

	Média Anual 2015	Média Anual 2016	Média Anual 2017
**UNIDADE TERAPIA INTENSIVA PÓS-OPERATÓRIA**			
Custo Médio do Leito-Dia	739,39	747,01	743,02
Custo Médio do Paciente-Dia	1.612,48	1.841,25	1.723,31
Custo Médio da Internação	4.192,44	5.339,62	4.739,10

Criado a partir do 3º trim/2014

	Média Anual 2015	Média Anual 2016	Média Anual 2017
**ENFERMARIA DE CLÍNICA CIRURGIA - UE** (Inclui: Cir.Urg., Ortop., C.C.P., Gineco., Semi Intens., Isolamento)			
Custo Médio do Leito-Dia	419,09	389,70	403,86
Custo Médio do Paciente-Dia	637,32	581,41	607,91
Custo Médio da Internação	2.485,55	2.441,91	2.462,03
**ENFERMARIA DE CLÍNICA MÉDICA - UE**			
Custo Médio do Leito-Dia	600,13	549,78	573,43
Custo Médio do Paciente-Dia	868,92	789,35	826,33
Custo Médio da Internação	4.952,85	4.736,10	4.834,02
**ENFERMARIA NEUROLOGIA - UE**			
Custo Médio do Leito-Dia	588,62	829,36	693,33
Custo Médio do Paciente-Dia	818,88	1.016,16	908,14
Custo Médio da Internação	6.796,73	7.722,83	7.219,74
**UNIDADE CORONARIANA - UE**			
Custo Médio do Leito-Dia	1.102,85	1.049,71	1.074,71
Custo Médio do Paciente-Dia	1.575,34	1.364,96	1.461,45
Custo Médio da Internação	8.191,76	7.507,28	7.818,75
**ENFERMARIA DE PEDIATRIA U.E.**			
Custo Médio do Leito-Dia	482,87	425,39	453,42
Custo Médio do Paciente-Dia	856,89	767,21	810,25
Custo Médio da Internação	5.141,32	5.063,61	5.104,55
**ENFERMARIA DE MI INFANTIL U.E.**			
Custo Médio do Leito-Dia	458,52	566,22	512,03
Custo Médio do Paciente-Dia	764,32	901,10	832,02
Custo Médio da Internação	6.802,43	7.298,87	7.072,13
**ENFERMARIA DE UNID.DE QUEIMADOS**			
Custo Médio do Leito-Dia	522,06	448,42	485,02
Custo Médio do Paciente-Dia	747,88	671,02	709,51
Custo Médio da Internação	11.218,20	10.669,25	10.961,96
**C.T.I. ADULTO - U.E.**			
Custo Médio do Leito-Dia	1.602,66	1.444,52	1.521,76
Custo Médio do Paciente-Dia	2.132,46	1.772,43	1.944,50
Custo Médio da Internação	25.163,02	22.864,40	24.014,52
**C.T.I. PEDIÁTRICO - U.E.**			
Custo Médio do Leito-Dia	1.331,88	1.255,37	1.292,70
Custo Médio do Paciente-Dia	2.131,78	2.079,22	2.104,73
Custo Médio da Internação	19.186,03	22.455,57	20.836,84

Seção de Custos, 12 de abril de 2018

A Assessoria Técnica, através da Seção de Custos, divulga o custo médio de alguns serviços prestados no HCRP, durante os anos de 2016 a 2018.

Custo Médio Atendimentos

	Anual 2016	Média Anual 2017	Anual 2018
Ambulatório	71,80	75,78	78,23
Ambulatório HC CRIANÇA	110,96	117,99	125,19
UETDI	204,41	201,85	242,37
CER - S. Médico Reabilitação	95,87	102,25	101,80
CEOF Otorrino	- x -	- x -	82,40
Casa 20	63,44	- x -	- x -
Sala de Urgência [1]	596,02	610,06	627,78
Pediatria - UE	510,90	558,29	505,95
Atendimento Queimados	126,36	136,20	65,46

Notas: (1) A Sala de Urgência engloba a Observação Adulto, Estabilização Clínica, G.O. Atendimento e Sala de Urgência.

Custo Médio da Hora

	Anual 2016	Média Anual 2017	Anual 2018
**Centro Cirúrgico**			
Central (sem Anestesia)	684,57	688,45	844,61
Unid.de Emergência (com Anestesia)	804,78	814,60	967,99
**Anestesiologia**			
Campus	430,05	430,29	503,63
**Recuperação**			
Campus	39,38	37,65	43,64
Unidade de Emergência	53,48	45,50	53,36
**Hemodiálise**			
Paciente crônico	266,00	242,00	322,00
Paciente agudo	399,00	363,00	483,00

Unidades de Internação

(não são incluídos no custo do paciente-dia Exames Laboratoriais e de Diagnóstico)

	Anual 2016	Média Anual 2017	Anual 2018
**TRANSPLANTE MEDULA OSSEA**			
Custo Médio do Leito-Dia	775,13	742,27	873,40
Custo Médio do Paciente-Dia	1.942,36	1.784,84	2.133,89
Custo Médio da Internação	30.883,52	28.021,99	34.142,24
**CLINICA MÉDICA 5º e 6º andares [1]**			
Custo Médio do Leito-Dia	256,87	263,09	292,33
Custo Médio do Paciente-Dia	516,88	530,69	585,57
Custo Médio da Internação	2.946,22	3.131,07	3.044,96

Notas: (1) A partir de 2012 somado a Unidade Metabólica e desmembrado a Derma e Imuno

(1) A partir do 2º trim/2014 desmembrado a Enfermaria Hemato-Químio

	Anual 2016	Média Anual 2017	Anual 2018
**CLINICA MÉDICA 4º andar (Derma e Imuno)**			
Custo Médio do Leito-Dia	256,48	270,96	289,28
Custo Médio do Paciente-Dia	583,74	637,23	670,02
Custo Médio da Internação	2.685,20	2.708,23	2.948,09

Notas: (1) Desmembrado da Cl. Médica a partir de 2012

	Anual 2016	Média Anual 2017	Anual 2018
**MOLESTIAS INFECTOCONTAGIOSA**			
Custo Médio do Leito-Dia	315,84	315,02	359,58
Custo Médio do Paciente-Dia	637,14	678,10	680,48
Custo Médio da Internação	6.435,11	7.221,77	5.784,08
**CLINICA CIRURGICA CAMPUS [1]**			
Custo Médio do Leito-Dia	281,36	303,89	295,75
Custo Médio do Paciente-Dia	590,79	623,28	619,21
Custo Médio da Internação	2.540,40	2.617,78	2.600,68

Notas: (1) Somado a Neurocirurgia a partir do 4º trim/2014

	Anual 2016	Média Anual 2017	Anual 2018
**UNIDADE DE TRANSPLANTE DE FÍGADO**			
Custo Médio do Leito-Dia	818,32	835,36	552,15
Custo Médio do Paciente-Dia	1.391,75	1.390,60	1.169,41
Custo Médio da Internação	6.958,75	6.257,70	5.613,17
**ENFERMARIA DE PSIQUIATRIA**			
Custo Médio do Leito-Dia	400,85	417,38	479,85
Custo Médio do Paciente-Dia	582,46	618,65	707,67
Custo Médio da Internação	5.824,60	5.877,18	6.935,17
**ENFERMARIA DE NEUROLOGIA**			
Custo Médio do Leito-Dia	279,34	287,31	351,31
Custo Médio do Paciente-Dia	558,76	581,83	690,96
Custo Médio da Internação	3.240,81	3.316,43	3.592,99
**ENFERMARIA DE ORTOPEDIA**			
Custo Médio do Leito-Dia	215,11	215,89	248,68
Custo Médio do Paciente-Dia	507,48	493,21	584,65
Custo Médio da Internação	2.892,64	2.811,30	3.332,51
**ENFERMARIA 12º ANDAR (OFT-ORL-CCP)**			
Custo Médio do Leito-Dia	201,07	219,64	224,46
Custo Médio do Paciente-Dia	575,94	581,42	650,43
Custo Médio da Internação	1.900,60	2.005,90	2.146,42
**ENFERMARIA DE GINECOLOGIA E OBSTETRÍCIA [1]**			
Custo Médio do Leito-Dia	209,72	203,19	317,07
Custo Médio do Paciente-Dia	413,05	410,41	900,88
Custo Médio da Internação	1.941,34	1.867,37	2.342,29

Notas: (1) a partir 2018 desmembrado Obstetrícia *(mudança prédio HC Criança)*

	Anual 2016	Média Anual 2017	Anual 2018
**ENFERMARIA OBSTÉTRICA**			
Custo Médio do Leito-Dia	- x -	- x -	282,21
Custo Médio do Paciente-Dia	- x -	- x -	473,99
Custo Médio da Internação	- x -	- x -	2.606,95
**ENFERMARIA DE PEDIATRIA CAMPUS**			
Custo Médio do Leito-Dia	443,96	448,79	528,05
Custo Médio do Paciente-Dia	990,42	959,01	1.057,86
Custo Médio da Internação	5.249,23	5.322,51	6.241,37
**C.T.I. PEDIÁTRICO CAMPUS**			
Custo Médio do Leito-Dia	1.127,47	1.172,00	1.091,10
Custo Médio do Paciente-Dia	1.857,23	1.919,04	2.260,56
Custo Médio da Internação	13.372,06	16.983,50	18.310,54

	Anual 2016	Média Anual 2017	Anual 2018
**UETDI - ENFERMARIA**			
Custo Médio do Leito-Dia	569,09	600,04	734,58
Custo Médio do Paciente-Dia	837,25	887,92	998,78
Custo Médio da Internação	10.381,90	11.542,96	12.784,38
**C.T.I. ADULTO CAMPUS**			
Custo Médio do Leito-Dia	1.688,28	1.737,64	1.728,29
Custo Médio do Paciente-Dia	2.529,94	2.608,30	2.847,24
Custo Médio da Internação	20.998,50	20.214,33	23.916,82
**UNIDADE CORONARIANA**			
Custo Médio do Leito-Dia	1.283,73	1.248,00	1.433,37
Custo Médio do Paciente-Dia	1.694,70	1.622,43	1.935,86
Custo Médio da Internação	11.523,96	9.410,09	12.195,92
**UTR** (2º ANDAR)			
Custo Médio do Leito-Dia	503,38	528,44	572,56
Custo Médio do Paciente-Dia	873,75	922,63	1.088,79
Custo Médio da Internação	5.941,50	5.628,04	6.859,38
**UNIDADE TERAPIA INTENSIVA NEONATAL**			
Custo Médio do Leito-Dia	857,53	878,82	973,33
Custo Médio do Paciente-Dia	1.200,64	1.221,22	1.450,74
Custo Médio da Internação	14.887,94	15.509,49	18.714,55
**UNIDADE CUIDADOS INTERMEDIARIOS NEONATAL - UCIN**			
Custo Médio do Leito-Dia	379,10	383,57	657,64
Custo Médio do Paciente-Dia	616,18	612,71	885,16
Custo Médio da Internação	2.587,96	2.573,38	11.418,56
**CIREP**			
Custo Médio do Leito-Dia	2.248,42	2.305,81	2.619,05
Custo Médio do Paciente-Dia	2.527,90	2.585,77	3.061,80
Custo Médio da Internação	10.111,60	10.213,79	12.859,56
**UNIDADE DE TERAPIA IMUNOLOGICA**			
Custo Médio do Leito-Dia	1.002,83	964,15	1.303,27
Custo Médio do Paciente-Dia	1.702,29	1.852,10	2.095,67
Custo Médio da Internação	7.319,85	7.408,40	23.681,07
**ENFERMARIA DE ONCOLOGIA**			
Custo Médio do Leito-Dia	436,52	452,59	469,51
Custo Médio do Paciente-Dia	794,00	830,43	869,12
Custo Médio da Internação	3.414,21	3.570,83	3.997,93
**ENFERMARIA HEMATO-QUÍMIO 6º ANDAR** [1]			
Custo Médio do Leito-Dia	494,02	515,20	558,86
Custo Médio do Paciente-Dia	1.343,55	1.424,42	1.461,79
Custo Médio da Internação	9.673,57	10.042,14	9.940,20

Notas: (1) Desmembrado da Cl. Médica a partir do 2º trim/2014

	Anual 2016	Média Anual 2017	Anual 2018
**UNIDADE TERAPIA INTENSIVA PÓS-OPERATÓRIA**			
Custo Médio do Leito-Dia	747,01	743,49	794,52
Custo Médio do Paciente-Dia	1.841,25	1.724,18	1.904,42
Custo Médio da Internação	5.339,62	4.741,48	4.761,05

	Anual 2016	Média Anual 2017	Anual 2018
**ENFERMARIA DE CLÍNICA CIRURGIA - UE** (Inclui: Cir.Urg., Ortop., C.C.P., Gineco., Semi Intens., Isolar			
Custo Médio do Leito-Dia	389,70	404,15	414,29
Custo Médio do Paciente-Dia	581,41	608,23	629,80
Custo Médio da Internação	2.441,91	2.463,35	2.708,12
**ENFERMARIA DE CLÍNICA MÉDICA - UE**			
Custo Médio do Leito-Dia	549,78	574,00	513,45
Custo Médio do Paciente-Dia	789,35	826,93	771,33
Custo Médio da Internação	4.736,10	4.837,54	4.627,98
**ENFERMARIA NEUROLOGIA - UE**			
Custo Médio do Leito-Dia	829,36	694,23	834,71
Custo Médio do Paciente-Dia	1.016,16	909,04	1.081,84
Custo Médio da Internação	7.722,83	7.226,91	8.005,62
**UNIDADE CORONARIANA - UE**			
Custo Médio do Leito-Dia	1.049,71	1.075,22	1.399,57
Custo Médio do Paciente-Dia	1.364,96	1.462,00	1.806,54
Custo Médio da Internação	7.507,28	7.821,72	9.574,68
**ENFERMARIA DE PEDIATRIA U.E.**			
Custo Médio do Leito-Dia	425,39	454,51	506,36
Custo Médio do Paciente-Dia	767,21	811,75	915,39
Custo Médio da Internação	5.063,61	5.114,04	4.760,03
**ENFERMARIA DE MI INFANTIL U.E.**			
Custo Médio do Leito-Dia	566,22	512,38	853,02
Custo Médio do Paciente-Dia	901,10	832,47	1.196,89
Custo Médio da Internação	7.298,87	7.076,00	12.567,36
**ENFERMARIA DE UNID.DE QUEIMADOS**			
Custo Médio do Leito-Dia	448,42	485,16	679,77
Custo Médio do Paciente-Dia	671,02	709,68	1.206,64
Custo Médio da Internação	10.669,25	10.964,59	17.858,27
**C.T.I. ADULTO - U.E.**			
Custo Médio do Leito-Dia	1.444,52	1.523,35	1.564,72
Custo Médio do Paciente-Dia	1.772,43	1.946,08	2.124,79
Custo Médio da Internação	22.864,40	24.034,06	26.772,35
**C.T.I. PEDIÁTRICO - U.E.**			
Custo Médio do Leito-Dia	1.255,37	1.293,51	1.367,97
Custo Médio do Paciente-Dia	2.079,22	2.105,80	2.405,12
Custo Médio da Internação	22.455,57	20.847,40	19.721,97

A Assessoria Técnica, através da Seção de Custos, divulga o custo médio de alguns serviços prestados no HCRP, nos anos de 2019 a 2022.

Cabe ressaltar que os custos dos anos de 2020 e 2021, dentre outros aspectos (como alteração/revisão critérios rateio), sofreram mais os impactos devido à pandemia da COVID-19.

* Custo Médio Atendimentos	Anual 2019	Anual 2020	Anual 2021	Anual 2022
Ambulatório	81,54	51,12	42,84	43,45
Ambulatório HC CRIANÇA	113,27	87,19	68,25	56,53
UETDI	267,47	92,81	79,31	83,48
CER - S. Médico Reabilitação	105,51	43,40	29,25	25,94
CEOF Otorrino	83,94	127,95	90,61	54,35
CER - AMB. ORTOPEDIA	39,56	41,43	31,69	25,84
Sala de Urgência [1]	687,58	510,05	474,55	467,89
Pediatria - UE	411,20	335,39	328,30	324,15
Atendimento Queimados	102,76	164,18	181,23	204,09
SEAVIDAS	181,55	171,89	123,33	118,65
Atend. Covid CP *(áreas amarela e azul)*	- x -	219,79	173,28	481,64
Atend. Covid CP *(área vermelha)*	- x -	373,63	- x -	- x -
Central de Quimioterapia Adulto	1.237,05	1.206,91	1.158,87	1.053,96
** Central de Quimioterapia Pediátrica	- x -	- x -	- x -	665,25

Notas: (1) A Sala de Urgência engloba a Observação Adulto, Estabilização Clínica, G.O. Atendimento e Sala de Urgência.

* A partir de 2020, sem valor referente às equipes médicas, exceto algumas áreas que possuem equipes específicas

** Desmembrado do Amb. HC Criança a partir 2º sem/2022

* Custo Médio Hora / Sessão	Anual 2019	Anual 2020	Anual 2021	Anual 2022
**Centros Cirúrgicos**				
Central Campus	952,02	862,98	753,55	776,93
Unid.de Emergência	920,04	625,40	561,43	614,94
**Anestesiologia**				
Anestesiologia	499,82	676,72	485,67	532,97
**Recuperação**				
Campus	68,49	85,73	68,49	57,27
Unidade de Emergência	50,14	70,58	64,28	51,07
**Hemodiálise** *(sessão)*				
Paciente crônico	340,00	366,00	336,00	382,00
Paciente agudo	510,00	549,00	504,00	573,00

* A partir de 2020, sem valor referente às equipes médicas, exceto algumas áreas que possuem equipes específicas

* Unidades de Internação	Anual 2019	Anual 2020	Anual 2021	Anual 2022
**TRANSPLANTE MEDULA OSSEA**				
Custo Médio do Leito-Dia	848,37	836,79	768,58	754,27
Custo Médio do Paciente-Dia	2.379,61	2.302,13	2.248,88	2.255,62
Custo Médio da Internação	41.881,14	43.740,47	55.322,45	42.405,66

(não são incluídos no custo do paciente-dia Exames Laboratoriais e de Diagnóstico)

* A partir de 2020, sem valor referente às equipes médicas, exceto algumas áreas que possuem equipes específicas

	Anual 2019	Anual 2020	Anual 2021	Anual 2022
**CLINICA MÉDICA 5º e 6º andares**				
Custo Médio do Leito-Dia	286,06	272,89	387,22	416,44
Custo Médio do Paciente-Dia	662,99	732,00	825,43	871,78
Custo Médio da Internação	3.049,75	3.513,60	4.127,16	4.358,91
*** CLINICA MÉDICA 4º andar**				
Custo Médio do Leito-Dia	307,63	2.214,55	-	-
Custo Médio do Paciente-Dia	691,04	1.829,26	-	-
Custo Médio da Internação	3.178,78	6.402,41	-	-

* Não internou nessa área em 2021 e 2022 (devido a pandemia Covid-19, passou a internar provisoriamente no 5º andar)

	Anual 2019	Anual 2020	Anual 2021	Anual 2022
**MOLESTIAS INFECTOCONTAGIOSA**				
Custo Médio do Leito-Dia	376,99	334,25	293,33	344,91
Custo Médio do Paciente-Dia	747,05	832,25	856,27	786,03
Custo Médio da Internação	7.096,98	7.573,48	9.333,34	9.353,76
**CLINICA CIRURGICA CAMPUS**				
Custo Médio do Leito-Dia	335,49	322,23	320,92	364,52
Custo Médio do Paciente-Dia	794,12	774,35	690,55	768,64
Custo Médio da Internação	2.541,18	2.787,66	2.762,20	3.382,02
**UNIDADE DE TRANSPLANTE DE FÍGADO**				
Custo Médio do Leito-Dia	456,87	623,33	553,87	616,64
Custo Médio do Paciente-Dia	1.148,44	1.597,04	1.299,32	1.387,02
Custo Médio da Internação	4.938,29	7.825,50	6.626,53	6.657,70
**ENFERMARIA DE PSIQUIATRIA**				
Custo Médio do Leito-Dia	510,39	422,50	485,63	589,28
Custo Médio do Paciente-Dia	766,46	649,23	707,83	906,79
Custo Médio da Internação	8.201,12	6.362,45	11.325,25	14.599,32
*** ENFERMARIA DE NEUROLOGIA**				
Custo Médio do Leito-Dia	380,01	887,90	-	-
Custo Médio do Paciente-Dia	878,67	2.115,23	-	-
Custo Médio da Internação	4.041,88	9.095,49	-	-

* Não internou nessa área em 2021 e 2022 (devido a pandemia Covid-19, passou a internar provisoriamente no 5º andar)

	Anual 2019	Anual 2020	Anual 2021	Anual 2022
**ENFERMARIA DE ORTOPEDIA**				
Custo Médio do Leito-Dia	320,86	282,28	348,87	372,50
Custo Médio do Paciente-Dia	679,04	738,05	744,95	732,03
Custo Médio da Internação	3.598,91	4.059,28	3.426,77	3.879,76
**ENFERMARIA 12º ANDAR (HOSPITAL SEMANA)**				
Custo Médio do Leito-Dia	291,78	659,06	489,07	436,63
Custo Médio do Paciente-Dia	617,50	1.514,91	1.084,32	847,19
Custo Médio da Internação	1.852,50	3.938,77	3.686,69	3.473,48
*** ENFERMARIA DE GINECOLOGIA ~~E OBSTETRÍCIA~~ [1]**				
Custo Médio do Leito-Dia	341,84	777,41	-	-
Custo Médio do Paciente-Dia	948,24	1.758,81	-	-
Custo Médio da Internação	3.034,37	6.331,72	-	-

* Não internou mais nessa área como Enf. Ginecologia, desde o ínicio da pandemia Covid-19 (a partir 2º trim/20)

Notas: (1) a partir 2018 desmembrado Obstetrícia *(mudança prédio HC Criança)*

	Anual 2019	Anual 2020	Anual 2021	Anual 2022
*** ENFERMARIA 8º ANDAR (HOSPITAL SEMANA)**				
Custo Médio do Leito-Dia	-	-	545,79	358,71
Custo Médio do Paciente-Dia	-	-	1.263,53	714,00
Custo Médio da Internação	-	-	3.916,94	2.998,80

* Voltou a internar a partir set/2021 (antiga área Enf. Ginecologia)

	Anual 2019	Anual 2020	Anual 2021	Anual 2022
**ENFERMARIA OBSTÉTRICA**				
Custo Médio do Leito-Dia	288,05	291,28	273,77	295,77
Custo Médio do Paciente-Dia	451,79	540,12	523,08	540,03
Custo Médio da Internação	2.484,85	2.646,59	2.615,40	2.808,16
**ENFERMARIA DE PEDIATRIA CAMPUS**				
Custo Médio do Leito-Dia	618,47	428,94	454,08	543,71
Custo Médio do Paciente-Dia	1.105,37	1.153,88	1.115,97	1.138,35
Custo Médio da Internação	7.627,05	7.038,67	5.803,04	7.626,95
**C.T.I. PEDIÁTRICO CAMPUS**				
Custo Médio do Leito-Dia	1.144,87	1.325,11	1.532,28	1.565,66
Custo Médio do Paciente-Dia	2.275,20	3.144,07	2.701,16	2.765,40
Custo Médio da Internação	18.429,12	24.209,34	19.988,58	25.994,76
*** UETDI - ENFERMARIA**				
Custo Médio do Leito-Dia	823,46	683,07	627,02	592,95
Custo Médio do Paciente-Dia	1.048,57	1.128,64	1.381,11	1.261,02
Custo Médio da Internação	14.050,84	11.737,86	14.915,99	7.818,32
* Voltou a internar pacientes Não Covid a partir out/2021				
*** CTI ADULTO CAMPUS**				
Custo Médio do Leito-Dia	1.610,81	1.377,36	1.136,98	1.777,62
Custo Médio do Paciente-Dia	2.374,88	2.831,79	2.476,88	3.037,75
Custo Médio da Internação	18.049,09	22.371,14	18.824,29	22.783,13
* Voltou a internar como CTI Adulto em set/21 [espaço utilizado como CTI Covid 2, devido à pandemia Covid-19 (início mar/20)]				
*** UNIDADE CORONARIANA**				
Custo Médio do Leito-Dia	1.380,38	1.655,64	1.359,11	1.608,86
Custo Médio do Paciente-Dia	1.746,42	2.551,78	2.105,26	2.373,52
Custo Médio da Internação	11.526,37	18.627,99	15.789,45	16.377,29
* Voltou a internar como UCO em nov/21 [espaço utilizado como CTI Covid 1, devido à pandemia Covid-19 (início mar/20)]				
**UNIDADE TRANSPLANTE RENAL (UTR)**				
Custo Médio do Leito-Dia	540,86	330,65	334,93	373,20
Custo Médio do Paciente-Dia	1.225,36	897,89	786,15	850,92
Custo Médio da Internação	8.332,45	4.938,40	5.660,28	5.530,98
**UNIDADE TERAPIA INTENSIVA NEONATAL**				
Custo Médio do Leito-Dia	978,18	668,41	637,65	758,96
Custo Médio do Paciente-Dia	1.579,76	1.327,03	1.098,52	1.184,00
Custo Médio da Internação	18.641,17	15.128,14	14.830,02	16.576,00
**UNIDADE CUIDADOS INTERMEDIARIOS NEONATAL - UCIN**				
Custo Médio do Leito-Dia	662,94	355,50	365,13	387,85
Custo Médio do Paciente-Dia	1.022,83	787,18	691,36	655,66
Custo Médio da Internação	10.432,87	7.793,08	6.913,60	8.785,84
**CIREP**				
Custo Médio do Leito-Dia	1.986,54	2.708,69	2.567,90	1.245,11
Custo Médio do Paciente-Dia	2.318,35	3.891,43	6.188,17	2.462,78
Custo Médio da Internação	12.287,26	17.900,58	24.752,68	12.067,62
*** UNIDADE DE TERAPIA IMUNOLOGICA**				
Custo Médio do Leito-Dia	1.327,00	1.758,23	-	-
Custo Médio do Paciente-Dia	2.245,82	3.275,06	-	-
Custo Médio da Internação	20.886,13	22.270,41	-	-
* Não internou nessa área desde o ínicio da pandemia Covid-19 (a partir 2º trim/20)				

	Anual 2019	Anual 2020	Anual 2021	Anual 2022
**UNIDADE DE ONCOLOGIA [1]**				
Custo Médio do Leito-Dia	430,26	386,36	318,11	-
Custo Médio do Paciente-Dia	832,81	934,19	799,14	-
Custo Médio da Internação	3.581,08	3.643,34	3.196,54	-

[1] Desmembrado em Onco Clínica e Cirúrgica a partir do 2º Sem/21

	Anual 2019	Anual 2020	Anual 2021	Anual 2022
**UNIDADE DE ONCOLOGIA CLÍNICA [1]**				
Custo Médio do Leito-Dia	-	-	489,80	664,83
Custo Médio do Paciente-Dia	-	-	1.195,24	1.666,35
Custo Médio da Internação	-	-	3.227,15	4.999,06

[1] Desmembrado da Unid. Oncologia a partir do 2º Sem/21

	Anual 2019	Anual 2020	Anual 2021	Anual 2022
**UNIDADE DE ONCOLOGIA CIRÚRGICA [1]**				
Custo Médio do Leito-Dia	-	-	288,09	363,10
Custo Médio do Paciente-Dia	-	-	770,94	720,24
Custo Médio da Internação	-	-	3.623,42	3.241,08

[1] Desmembrado da Unid. Oncologia a partir do 2º Sem/21

	Anual 2019	Anual 2020	Anual 2021	Anual 2022
**ENFERMARIA HEMATO-QUÍMIO 6º ANDAR [1]**				
Custo Médio do Leito-Dia	484,19	387,27	373,61	436,49
Custo Médio do Paciente-Dia	1.291,96	1.069,06	1.044,30	1.079,36
Custo Médio da Internação	9.431,29	8.552,49	9.816,42	8.850,73

Notas: (1) Desmembrado da Cl. Médica a partir do 2º trim/2014

	Anual 2019	Anual 2020	Anual 2021	Anual 2022
**UNIDADE TERAPIA INTENSIVA PÓS-OPERATÓRIA**				
Custo Médio do Leito-Dia	901,11	677,79	639,25	796,03
Custo Médio do Paciente-Dia	1.916,28	1.647,98	1.495,19	1.840,86
Custo Médio da Internação	4.790,69	4.943,95	6.728,34	6.074,82

	Anual 2019	Anual 2020	Anual 2021	Anual 2022
*** CTI NÃO COVID CAMPUS**				
Custo Médio do Leito-Dia	- x -	1.486,70	1.087,62	-
Custo Médio do Paciente-Dia	- x -	2.414,34	1.825,38	-
Custo Médio da Internação	- x -	13.037,43	11.864,98	-

* Área desativada a partir nov/2021

	Anual 2019	Anual 2020	Anual 2021	Anual 2022
**UETDI ENFERMARIA COVID**				
Custo Médio do Leito-Dia	- x -	619,41	552,89	667,14
Custo Médio do Paciente-Dia	- x -	1.563,47	1.376,54	1.312,12
Custo Médio da Internação	- x -	7.973,67	8.626,32	7.872,75

	Anual 2019	Anual 2020	Anual 2021	Anual 2022
*** CTI COVID 1 CAMPUS**				
Custo Médio do Leito-Dia	- x -	1.120,50	1.597,54	-
Custo Médio do Paciente-Dia	- x -	2.771,00	3.077,61	-
Custo Médio da Internação	- x -	26.324,53	31.699,42	-

* Área desativada a partir set/2021

	Anual 2019	Anual 2020	Anual 2021	Anual 2022
*** CTI COVID 2 CAMPUS**				
Custo Médio do Leito-Dia	- x -	1.544,94	1.381,55	1.716,54
Custo Médio do Paciente-Dia	- x -	2.767,77	2.979,30	3.333,81
Custo Médio da Internação	- x -	42.623,60	39.028,81	21.336,41

* Reativado 1 mês em 2022; área desativada a partir set/2021

	Anual 2019	Anual 2020	Anual 2021	Anual 2022
**CTI COVID 3 CAMPUS**				
Custo Médio do Leito-Dia	- x -	1.004,85	1.305,90	1.857,77
Custo Médio do Paciente-Dia	- x -	2.798,18	3.112,58	3.588,37
Custo Médio da Internação	- x -	28.541,44	32.993,35	24.400,93

	Anual 2019	Anual 2020	Anual 2021	Anual 2022
*** CTI COVID 4 CAMPUS**				
Custo Médio do Leito-Dia	- x -	1.035,19	1.357,74	-
Custo Médio do Paciente-Dia	- x -	3.315,31	3.103,87	-
Custo Médio da Internação	- x -	33.816,17	37.867,26	-

* Área desativada a partir 4º trim/2021

	Anual 2019	Anual 2020	Anual 2021	Anual 2022
**ENFERMARIA DE CLÍNICA CIRURGIA - UE** (Inclui: Cir.Urg., Ortop., C.C.P., Gineco., Semi Intens., Isolamento)				
Custo Médio do Leito-Dia	435,03	358,42	328,22	361,60
Custo Médio do Paciente-Dia	642,75	642,92	592,31	624,91
Custo Médio da Internação	2.828,08	2.507,39	2.487,69	2.999,58
**ENFERMARIA DE CLÍNICA MÉDICA - UE**				
Custo Médio do Leito-Dia	476,05	474,14	472,26	536,19
Custo Médio do Paciente-Dia	747,46	853,35	885,70	951,67
Custo Médio da Internação	3.961,51	3.925,43	4.959,91	5.995,54
*** ENFERMARIA NEUROLOGIA - UE**				
Custo Médio do Leito-Dia	817,30	594,13	577,76	689,41
Custo Médio do Paciente-Dia	1.095,66	962,94	953,94	1.161,53
Custo Médio da Internação	7.012,23	6.836,89	7.631,55	12.079,93

* Desmembrado Unid. AVC a partir 2º sem/2022

	Anual 2019	Anual 2020	Anual 2021	Anual 2022
*** UNIDADE DE AVC - UE**				
Custo Médio do Leito-Dia	- x -	- x -	- x -	637,82
Custo Médio do Paciente-Dia	- x -	- x -	- x -	933,57
Custo Médio da Internação	- x -	- x -	- x -	9.522,46

* Desmembrado Enfermaria Neurologia UE a partir 2º sem/2022

	Anual 2019	Anual 2020	Anual 2021	Anual 2022
**UNIDADE CORONARIANA - UE**				
Custo Médio do Leito-Dia	1.373,88	1.252,79	1.021,62	1.111,20
Custo Médio do Paciente-Dia	1.697,27	1.767,36	1.557,29	1.520,45
Custo Médio da Internação	8.825,78	9.543,75	8.720,83	8.362,49
**ENFERMARIA DE PEDIATRIA U.E.**				
Custo Médio do Leito-Dia	549,18	440,19	498,77	582,53
Custo Médio do Paciente-Dia	949,95	988,54	863,04	960,21
Custo Médio da Internação	4.369,76	5.931,24	3.193,24	5.185,15
**ENFERMARIA DE MI INFANTIL U.E.**				
Custo Médio do Leito-Dia	840,63	526,56	468,72	515,23
Custo Médio do Paciente-Dia	1.264,25	1.697,93	1.177,69	875,67
Custo Médio da Internação	9.861,18	4.754,22	2.473,15	3.940,53
**ENFERMARIA DE UNID.DE QUEIMADOS**				
Custo Médio do Leito-Dia	1.159,58	866,29	762,14	624,22
Custo Médio do Paciente-Dia	1.484,03	1.598,82	1.433,94	1.288,25
Custo Médio da Internação	20.776,41	20.944,51	19.214,74	18.421,93
**C.T.I. ADULTO - U.E.** [1]				
Custo Médio do Leito-Dia	1.513,56	1.328,66	1.245,90	1.441,63
Custo Médio do Paciente-Dia	2.165,50	2.335,33	2.308,31	2.515,69
Custo Médio da Internação	27.934,93	28.491,08	31.162,13	36.980,60

[1] Desmembrado CTI Covid UE em 2021

	Anual 2019	Anual 2020	Anual 2021	Anual 2022
**C.T.I. PEDIÁTRICO - U.E.**				
Custo Médio do Leito-Dia	2.630,52	1.817,48	1.493,26	1.774,83
Custo Médio do Paciente-Dia	4.066,43	4.064,21	2.968,75	3.095,21
Custo Médio da Internação	24.398,61	21.540,33	16.031,23	19.499,80

	Anual 2019	Anual 2020	Anual 2021	Anual 2022
**C.T.I. COVID - U.E. [1]**				
Custo Médio do Leito-Dia	- x -	- x -	1.957,38	-
Custo Médio do Paciente-Dia	- x -	- x -	3.786,02	-
Custo Médio da Internação	- x -	- x -	56.411,66	-

[1] Desmembrado CTI Adulto UE em 2021. Área desativada a partir ago/21

	Anual 2019	Anual 2020	Anual 2021	Anual 2022
**ATENDIMENTO COVID ÁREA VERMELHA - U.E. [1]**				
Custo Médio do Leito-Dia	- x -	- x -	529,28	-
Custo Médio do Paciente-Dia	- x -	- x -	1.670,24	2.278,88
Custo Médio da Internação	- x -	- x -	3.674,54	3.874,10

[1] Área desativada a partir 4º trim/22. Desmembrado da Sala Urgência em 2021
