# Supplementary material for: Understanding traumatic brain injury in elderly populations: a developing country perspective
Source: Clinics (Sao Paulo). 2026 Jul 28;81:101054. doi: 10.1016/j.clinsp.2026.101054 (PMC13446349; doi:10.1016/j.clinsp.2026.101054)
Supplement: Supplementary file 2 [file mmc1.docx]

**CLINICS-D-25-01150**

**Supplementary Material**

**Table** Primary and secondary injuries with respective ICD-10 codes.

| **Primary Injury** | **ICD-10** |
| --- | --- |
| Extracranial Injuries | S01, S02, S03, S05, S08, S09 |
| Subdural hemorrhage | S065 |
| Cerebral concussion | S060 |
| Focal brain trauma | S063 |
| Traumatic cerebral edema | S061 |
| Traumatic subarachnoid hemorrhage | S066 |
| Others Intracranial Injuries | S068, S069 |
| Epidural hematoma | S064 |
| Diffuse brain trauma | S062 |
| Intracranial trauma with prolonged coma | S067 |
|  |  |
| **Secondary Injury** | **ICD-10** |
| Thorax | S202, S220, S222, S223, S224, S411, S529 |
| Abdomen, Lumbar, and Pelvis | S270, S271, S272, S299, S300, S308, S309, S320, S322, S323, S324, S325, S327, S328, S363, S373, S399, S400 |
| Upper Limbs | S420, S421, S422, S423, S424 S430, S431, S460, S499, S501, S507, S509, S518, S519, S520, S521, S523, S525, S526, S531, S599, S610, S618, S620, S621, S623, S625, S626, S628, S631 |
| Lower Limbs | S700, S711, S720, S721, S724, S729, S798, S799, S800, S808, S810, S818, S820, S821, S824, S825, S826, S827, S828, S836, S868, S889, S899, S901, S903, S911, S913, S920, S923, S924, S925, S934 |
| Other Injuries | T07, T202, T212, T220, T222, T232, T242, T131, T139, T140, T200 |

Supplementary Material - PDF
